# A novel model forecasting perioperative red blood cell transfusion

**DOI:** 10.1038/s41598-022-20543-7

**Published:** 2022-09-27

**Authors:** Yawen Zhang, Xiangjie Fu, Xi Xie, Danyang Yan, Yanjie Wang, Wanting Huang, Run Yao, Ning Li

**Affiliations:** Department of Blood Transfusion, National Clinical Research Center for Geriatric Disorders, Xiangya Hospital, Clinical Transfusion Research Center, Central South University, Hunan Province, 87 Xiangya Road, Changsha, 410008 China

**Keywords:** Nomograms, Risk factors

## Abstract

We aimed to establish a predictive model assessing perioperative blood transfusion risk using a nomogram. Clinical data for 97,443 surgery patients were abstracted from the DATADRYAD website; approximately 75% of these patients were enrolled in the derivation cohort, while approximately 25% were enrolled in the validation cohort. Multivariate logical regression was used to identify predictive factors for transfusion. Receiver operating characteristic (ROC) curves, calibration plots, and decision curves were used to assess the model performance. In total, 5888 patients received > 1 unit of red blood cells; the total transfusion rate was 6.04%. Eight variables including age, race, American Society of Anesthesiologists' Physical Status Classification (ASA-PS), grade of kidney disease, type of anaesthesia, priority of surgery, surgery risk, and an 18-level variable were included. The nomogram achieved good concordance indices of 0.870 and 0.865 in the derivation and validation cohorts, respectively. The Youden index identified an optimal cut-off predicted probability of 0.163 with a sensitivity of 0.821 and a specificity of 0.744. Decision curve (DCA) showed patients had a standardized net benefit in the range of a 5–60% likelihood of transfusion risk. In conclusion, a nomogram model was established to be used for risk stratification of patients undergoing surgery at risk for blood transfusion. The URLs of web calculators for our model are as follows: http://www.empowerstats.net/pmodel/?m=11633_transfusionpreiction.

## Introduction

Blood transfusion is an essential life-saving treatment that is particularly suitable for surgical patients. In the United States, more than 13 million units of red blood cells (RBCs) are transfused clinically each year; the majority of these are used for surgery^1^. The selection of blood donors is becoming stricter, and people are simultaneously less interested in voluntary blood donation^2^; the combination of these factors has led to substantial blood shortages. Especially due to the global COVID-19 outbreak, blood shortage caused by a decrease in blood donations has become an important global public health concern that needs to be solved urgently^3^. Additionally, preoperative testing for surgeries often includes blood typing and antibody screening (T&S). However, most patients are not transfused after all^4^. Transfusion improves patients' ischemia and hypoxia symptoms and reduces mortality, though it may also cause adverse transfusion reactions (such as hemolytic reactions, transfusion-associated circulatory overload, transfusion-related acute lung injuries, and blood-borne infections)^5^. In the current study, we aimed to develop a blood transfusion prediction model for identifying high risk patients prior to surgery. Preoperative knowledge regarding high-risk transfusion patients might improve blood resource management, inform clinicians of the likelihood of postoperative morbidities, reduce costs by avoiding unnecessary preoperative typing expenditures, and more effectively triage patients unlikely to need a transfusion.

In prior research, most models have been developed in order to predict patients’ surgical transfusion needs for a single type of operation, such as for gynecologic surgery^6^, liver surgery^7^, or spine fusion surgery^8^. There is little study on the model forecasting red blood cell transfusion for normal perioperative patients. According to current medical guidelines^9^, surgical risk is divided into low risk, moderate risk, and high risk categories according to the difficulty and complexity of the operation. In addition, surgical patients have been divided into six grades within the American Society of Anesthesiologists scoring system according to patients' physical status prior to anaesthesia^10^. This combination of surgical risk grades and The American Society of Anesthesiologists' Physical Status Classification (ASA-PS) scores can provide reasonable classifications informing medical decision-making within operations.

In this study, we combined blood routine and clinical parameters, including surgical risk grades and ASA-PS scores, in order to construct a predictive model for blood transfusion in perioperative patients via secondary analysis of a large dataset. We hypothesized that this model would predict the necessity for RBCs transfusion reliably and effectively and thus help identify patients that might benefit most from a patient blood management (PBM) program.

## Methods

### Data resources and patients

Data were obtained from the 'DATADRYAD' database (available at www.datadryad.org). This website allows users to freely download raw data. According to the Dryad Terms of Service, we cited the Dryad data package in the present study (Sim et al. 2018: Data for prevalence of preoperative anemia, abnormal mean corpuscular volume, and red cell distribution width among surgical patients in Singapore, and their influence on one year mortality. Available at 10.5061/dryad.5772v). Variables included in the database file were as follows: age, gender, race, ASA-PS, cerebrovascular accidents, ischemic heart disease, congestive heart failure, diabetes mellitus, grade of kidney disease, type of anaesthesia, priority of surgery, surgery risk, anemia, mean corpuscular volume (MCV), red cell distribution width (RDW) and transfusion intra- and post-operation. 97,443 patients aged ≥ 18 years who underwent cardiac and non-cardiac surgeries (except for transplantation, minor surgeries, and burning operations) under anesthesia between 1 January 2012 and 31 October 2016 at Singapore General Hospital. Institutional review board approval was obtained (Singhealth CIRB2014/651/D) and all participants provided their written informed consent. In addition, we retrospective collected clinical data among 400 patients aged ≥ 18 years who underwent surgeries (except for transplantation, minor surgeries, and burning operations) using systematic random sampling^11^ between 1 March 2021 and 30 June 2021 at Xiangya Hospital of Central South University. Surgical disciplines that were included cardiothoracic, orthopaedics, obstetrics and gynaecology, general surgery, otolaryngorhinology, hand surgery, neurosurgery, colorectal surgery, urology, plastic surgery, and oromaxillofacial surgery. Clinical data of these patients included age, race, ASA-PS, grade of kidney disease, type of anaesthesia, priority of surgery, surgery risk, anemia, MCV, RDW and transfusion outcomes intra- and post-operation. These surgical patients completed pre-transfusion testing, including T&S, cross-matching (anti-human globulin microcolumn method) and transfusion infectious disease testing, prior to surgery. For elective surgical patient, after evaluating preoperative anemia, coagulation function and the amount of intraoperative blood loss by surgeon, blood bank determined eligibility for T&S and cross-matching based on the composition and quantity of blood requested by surgeon, then prepared the blood and preserved for 72 h. The Medical Ethics Committee of Xiangya Hospital of Central South University approved the sub-study (approval number: 202108852) and waived the informed consent for individual patients sowing to the retrospective nature of the study. This study was conducted in accordance with the principles of the Declaration of Helsinki and its later amendments.

All the included patients underwent a thorough physical examination and routine preoperative laboratory measurements at our institute. Following procedures reported in the previous literature^12^ we evaluated all surgical patients for possible blood transfusion. For the preoperative management of patients with anemia, we based on recommendations of American Association of Blood Banks (AABB)^13^ and expert consensus^14^. Transfusion was implemented when the haemoglobin level was lower than 70 g/L. For patients with haemoglobin between 70 and 100 g/L, decisions regarding RBCs transfusion should be based on their age, clinical signs, cardiac and pulmonary compensation, metabolic rate, and the presence of active bleeding, etc. For non-urgent surgery patients with moderate/severe anemia, surgery needs to be postponed until anemia is corrected.

### Definitions

Based on WHO standards^15^, anemia was classified as mild (Hb: 11.0–12.9 g/dL for men, 11–11.9 g/dL for women), moderate (Hb: 8–10.9 g/dL for both genders), or severe anemia (Hb: < 8.0 g/dL for both genders). According to the relevant literature^16^, RDW > 15.7% was defined as high RDW, whereas values ranging between 10.9 and 15.7% were defined as normal RDW. MCV > 100 fl was defined as high MCV, while 80–100 fl was defined as normal MCV and < 80 fl was defined as low MCV. Surgical risk and surgery priority classifications were based on the 2014 European Society of Cardiology and European Society of Anesthesiology guidelines^9^. The surgical risk was divided into low risk, moderate risk and high risk according to the difficulty and complexity of the operation and listed in Table 1S. ASA-PS recommendations were followed for the ASA-PS definitions^10^. Specifically, the ASA score was divided into six grades and listed in Table 2S. The grade of basic nephropathy before surgery will be graded according to Kidney Disease: Improving Global Outcomes guidelines^17^ and the glomerular filtration rate.

Perioperative blood transfusion, defined as ≥ 1 unit of red blood cells were accepted during surgery and within 30 days after surgery, was regarded as the outcome variable. Data regarding the transfusion of other blood components was not available in this study^16^.

### Statistical analyses

Patients who received 0 units RBCs transfusion were divided into the non-transfusion group, received 1 unit and 2 or more than 2 units of RBCs transfusion were divided into the transfusion group. We first compared the baseline data for patients in the transfusion and non-transfusion groups. Categorical variables were described as counts (percentages) and differences were assessed using Fisher's test. Secondly, the dataset was randomly divided in a 3:1 ratio, with the former participants used as the derivation cohort and the latter participants used as the validation cohort. To assess for interaction between Anemia and MCV/RDW, we calculated ORs comparing patients with none, mild, moderate/severe anemia after stratifying by MCV/RDW status. We did further subgroup analysis of the anemia categories stratified by MCV and RDW. We compared differences between the derivation and validation cohorts. Thirdly, univariate and multivariate logistic regression analyses were performed to explore associations between perioperative variables and blood transfusion risk in the derivation cohorts. According to the results of multivariate logistic regression analysis, we used Empower Stats software to formulate a nomogram. The nomogram can proportionally convert each regression coefficient in the logistic regression to a scale of 0 to 100 points^18^. The points of each independent variable were summed, and the predicted probabilities were derived from the total points. Fourthly, model performance was assessed via discrimination and calibration in the derivation and validation cohorts. A receiver operating characteristics (ROC) curve was used to evaluate the distinguishing ability of the model. Youden index (sensitivity + specificity − 1) was applied to determine optimal cutoff values. A calibration curve was used to evaluate the relationship between the predicted and actual probability. A decision curve was used to evaluate the clinical application value of the model. Briefly, the decision curve is a form of decision analysis that informs clinicians regarding the threshold probability range for predicting the clinical value of a model. Decision curve analysis determines the association between the selected prediction probability threshold and the relative value of false-positive and false-negative results in order to obtain the net benefit of using the model under this threshold. Then we validated the clinical application of this model at our institution. According to the formula for calculating the sample size: n ≥ (k/β)2P(1 − P). The estimated objective difference does not exceed 2% (β = 0.02), α = 0.05, one-side: k0.05 = 1.645, the probability of blood transfusion in the model is 6.04% (P = 6.04%). And we calculated sample size n ≥ 384. We collected variables according to predictors of model. Each variable corresponded a point which accorded to nomogram. Assigned points for all variables are then summed and can be got total points. ROC curve and cut-off value were used to assess the ability of total points in predicting transfusion risk of our institution. In addition, we obtained the percentage of benefited population by comparing the coincidence of predicted transfusion events with actual transfusion events.

All statistical analyses were performed using R software (The R Project for Statistical Computing, http://www.R-project.org; Vienna, Austria) and Empower Stats software (www.empowerstats.com, X&Y Solutions, Inc.).

## Results

### Demographic and clinical parameters

A total of 97,443 patients were included in the current analysis. In this study, 5888 patients received RBCs transfusion, with a final blood transfusion rate of 6.04%. The baseline demographic data and risk factors among patients with and without transfusion are summarized in Table 1. There were considerable differences between the transfusion and non-transfusion groups in terms of age, gender, race, ASA-PS, cerebrovascular accidents, ischemic heart disease, congestive heart failure, diabetes mellitus on insulin, grade of kidney disease, type of anesthesia, priority of surgery, surgery risk, anemia, MCV, and RDW.

**Table 1:** Baseline of patients in no transfusion and transfusion group.

	Total (n = 97,443)	No transfusion (n = 91,555)	Transfusion (n = 5888)	P-value
**Age (years)**				< 0.001
18–29	11,263 (11.56%)	10,834 (11.83%)	429 (7.29%)	
30–49	28,106 (28.84%)	26,970 (29.46%)	1136 (19.29%)	
50–69	41,475 (42.56%)	38,930 (42.52%)	2545 (43.22%)	
≥ 70	16,599 (17.03%)	14,821 (16.19%)	1778 (30.20%)	
**Gender**				< 0.001
Female	50,632 (51.96%)	47,343 (51.71%)	3289 (55.86%)	
Male	46,811 (48.04%)	44,212 (48.29%)	2599 (44.14%)	
**Race**				< 0.001
Chinese	69,485 (71.31%)	65,130 (71.14%)	4355 (73.96%)	
Malay	9826 (10.08%)	9252 (10.11%)	574 (9.75%)	
Indian	8621 (8.85%)	8263 (9.03%)	358 (6.08%)	
Others	9494 (9.74%)	8895 (9.72%)	599 (10.17%)	
**ASA-PS**				< 0.001
ASA 1	22,147 (22.73%)	21,558 (23.55%)	589 (10.00%)	
ASA 2	50,252 (51.57%)	47,910 (52.33%)	2342 (39.78%)	
ASA 3	17,793 (18.26%)	15,583 (17.02%)	2210 (37.53%)	
ASA 4–6	2150 (2.21%)	1691 (1.85%)	459 (7.80%)	
**Cerebrovascular accidents**				< 0.001
No	65,097 (66.81%)	61,194 (66.84%)	3903 (66.29%)	
Yes	1811 (1.86%)	1614 (1.76%)	197 (3.35%)	
**Ischemic heart disease**				< 0.001
No	59,953 (61.53%)	56,572 (61.79%)	3381 (57.42%)	
Yes	6712 (6.89%)	6008 (6.56%)	704 (11.96%)	
**Congestive heart failure**				< 0.001
No	67,619 (69.39%)	63,568 (69.43%)	4051 (68.80%)	
Yes	1413 (1.45%)	1233 (1.35%)	180 (3.06%)	
**Diabetes mellitus on insulin**				< 0.001
No	66,012 (67.74%)	62,083 (67.81%)	3929 (66.73%)	
Yes	2329 (2.39%)	2076 (2.27%)	253 (4.30%)	
**Grade of kidney disease**				< 0.001
G1	50,276 (51.60%)	47,496 (51.88%)	2780 (47.21%)	
G2	25,640 (26.31%)	24,263 (26.50%)	1377 (23.39%)	
G3	5850 (6.00%)	5159 (5.63%)	691 (11.74%)	
G4–5	3477 (3.57%)	2972 (3.25%)	505 (8.58%)	
**Type of anesthesia**				< 0.001
General anesthesia	83,100 (85.28%)	77,769 (84.94%)	5331 (90.54%)	
Regional/spinal anesthesia	14,343 (14.72%)	13,786 (15.06%)	557 (9.46%)	
**Priority of surgery**				< 0.001
Elective	78,161 (80.21%)	73,788 (80.59%)	4373 (74.27%)	
Emergency	19,282 (19.79%)	17,767 (19.41%)	1515 (25.73%)	
**Surgery risk**				< 0.001
Low	48,002 (49.26%)	47,350 (51.72%)	652 (11.07%)	
Moderate	39,724 (40.77%)	36,261 (39.61%)	3463 (58.81%)	
High	4626 (4.75%)	3364 (3.67%)	1262 (21.43%)	
**Anaemia**				< 0.001
None	67,099 (68.86%)	65,338 (71.36%)	1761 (29.91%)	
Mild	14,189 (14.56%)	13,103 (14.31%)	1086 (18.44%)	
Moderate/severe	11,597 (11.90%)	8698 (9.50%)	2899 (49.24%)	
**MCV**				< 0.001
80–100 normocytosis	78,501 (80.56%)	74,358 (81.22%)	4143 (70.36%)	
< 80 microcytosis	9840 (10.10%)	8675 (9.48%)	1165 (19.79%)	
> 100 macrocytosis	1369 (1.40%)	1194 (1.30%)	175 (2.97%)	
**RDW**				< 0.001
≤ 15.7	80,695 (82.81%)	76,906 (84.00%)	3789 (64.35%)	
> 15.7	8994 (9.23%)	7301 (7.97%)	1693 (28.75%)	

### Interactions between RDW, MCV and anemia

**Figure 1:**
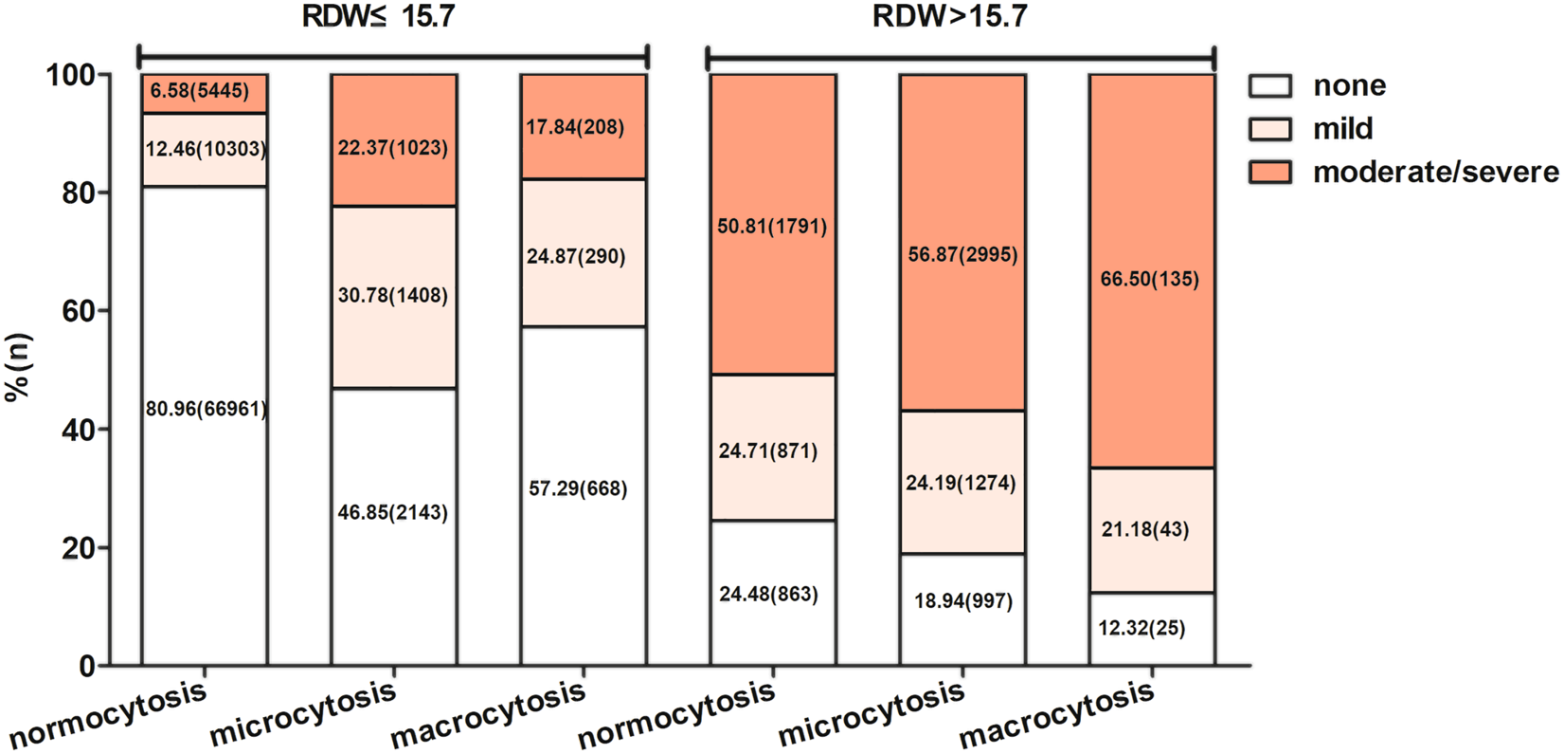
Distribution of anemia patients stratified by red cell distribution width (RDW) and mean corpuscular volume (MCV).

To assess the multiplicative effects of RDW by MCV and anemia status, logistic regression analysis was performed to explore the association between anemia and transfusion risk after stratifying by RDW and MCV. Table 2 shows the interaction between RDW (p for interaction = 0.002), MCV (p for interaction = 0.008), and anemia associated with transfusion risk. When the RDW value was ≤ 15.7%, the moderate/severe anemia rate with 6.58% was lower than none anemia (80.96%) and mild anemia (12.46%) for normocytosis, and similar results were observed for microcytosis, and macrocytosis. When the RDW value was > 15.7%, the moderate/severe anemia rate with 50.81% was higher than none anemia (24.48%) and mild anemia (24.71%) for normocytosis, and similar results were observed for microcytosis, and macrocytosis (Fig. 1).

**Table 2:** Interaction between RDW, MCV and anemia associated with transfusion risk.

Subgroup	Anemia (OR 95% CI)			P for interaction
	None	Mild	Moderate/severe	
**MCV**				0.008
80–100 normocytosis	1.0 (ref.)	3.25 (2.99, 3.53)	12.75 (11.88, 13.68)	
< 80 microcytosis	0.99 (0.79, 1.24)	2.13 (1.79, 2.53)	11.23 (10.30, 12.25)	
> 100 macrocytosis	1.85 (1.3, 2.63)	4.62 (3.27, 6.52)	16.29 (12.89, 20.6)	
**RDW**				0.002
≤ 15.7	1.0 (ref.)	3.04 (2.8, 3.3)	10.69 (9.92, 11.51)	
> 15.7	1.88 (1.51, 2.33)	3.46 ((2.96, 4.05)	15.17 (14.04, 16.39)

Because the interaction term between RDW, MCV, and anemia categories was statistically significant, we performed further subgroup analysis with respect to an 18-level variable stratified by RDW, MCV, and anemia severity.

### Model specifications and predictors of transfusion

The cases enrolled in the current study were divided into a derivation cohort (n = 73,082) and a validation cohort (n = 24,361) in a 3:1 ratio via a simple random sampling method. The baseline data for the derivation and validation cohorts are presented in Supplementary Table 3S; there were no statistically significant differences between the derivation and validation cohorts.

The following demographic and clinically relevant preoperative predictors were selected as candidate variables for the prediction model based on statistically significant associations observed in the univariate analysis within the derivation cohort (Table 3). Specifically, 11 variables that were strongly associated with blood transfusion and were included in the final multivariable analysis: age, gender, race, ASA-PS, ischemic heart disease, diabetes mellitus, grade of kidney disease, type of anesthesia, priority of surgery, surgery risk, and the 18-level variable defined above. On multivariate logistic regression analysis, eight variables, including age, race, ASA-PS, grade of kidney diseases, type of anesthesia, priority of surgery, surgery risk, and the 18-level variable, were independently associated with a statistically significantly increased odds of blood transfusion and were thus enrolled in the final nomogram model.

**Table 3:** Univariate and Multivariate logistic regression analysis in Derivation cohort.

Exposure	Univariate OR (95% CI)	Multivariate OR (95% CI)
**Age (years)**		
18–29	Reference	Reference
30–49	1.06 (0.93, 1.21)	0.72 (0.63, 0.84)*
50–69	1.65 (1.46, 1.86)*	0.88 (0.77, 1.02)
≥ 70	3.03 (2.67, 3.43)*	1.07 (0.92, 1.25)
**Gender**		
Female	Reference	Reference
Male	0.85 (0.80, 0.90)*	0.96 (0.89, 1.03)
**Race**		
Chinese	Reference	Reference
Malay	0.93 (0.84, 1.03)	0.87 (0.78, 0.98)*
Indian	0.69 (0.61, 0.78)*	0.77 (0.67, 0.89)*
Others	1.02 (0.92, 1.13)	1.18 (1.05, 1.32)*
**ASA-PS**		
ASA1	Reference	Reference
ASA 2	1.82 (1.64, 2.03)*	1.11 (0.99, 1.25)
ASA 3	5.19 (4.66, 5.78)*	1.61 (1.41, 1.85)*
ASA 4–6	9.92 (8.52, 11.54)*	1.96 (1.63, 2.37)*
**Cerebrovascular accidents**		
No	Reference	Reference
Yes	1.02 (0.96, 1.09)	1.05 (0.92, 1.19)
**Ischemic heart disease**		
No	Reference	Reference
Yes	1.92 (1.75, 2.12)*	0.98 (0.91, 1.05)
**Congestive heart failure**		
No	Reference	Reference
Yes	1.02 (0.96, 1.09)	0.94 (0.82, 1.07)
**Diabetes mellitus on insulin**		
No	Reference	Reference
Yes	1.90 (1.63, 2.21)*	0.85 (0.71, 1.02)
**Grade of kidney disease**		
G1	Reference	Reference
G2	0.90 (0.84, 0.96)*	0.89 (0.82, 0.96)*
G3	2.28 (2.06, 2.53)*	1.00 (0.88, 1.13)
G4-5	3.00 (2.67, 3.37)*	0.83 (0.71, 0.96)*
**Type of anesthesia**		
General anesthesia	Reference	Reference
Regional/spinal anesthesia	0.59 (0.54, 0.66)*	0.45 (0.40, 0.50)*
**Priority of surgery**		
Elective	Reference	Reference
Emergency	1.42 (1.33, 1.53)*	1.17 (1.08, 1.28)*
**Surgery risk**		
Low	Reference	Reference
Moderate	7.15 (6.49, 7.88)*	6.21 (5.61, 6.87)*
High	27.62 (24.58, 31.03)*	19.62 (17.28, 22.28)*
**18 level variables**		
0-Normal RDW, No anemia, Normal MCV	Reference	Reference
1-High RDW, Mod/Severe anemia, High MCV	29.97 (20.16, 44.55)*	30.92 (19.54, 48.90)*
2-High RDW, Mod/Severe anemia, Low MCV	14.45 (12.92, 16.17)*	13.63 (12.04, 15.42)*
3-High RDW, Mild anemia, High MCV	5.23 (1.57, 17.39)*	4.57 (1.31, 16.01)*
4-High RDW, Mild anemia, Low MCV	2.44 (2.17, 2.76)*	2.38 (2.08, 2.72)*
5-Normal RDW, Mod/Severe anemia, High MCV	13.71 (9.54, 19.70)*	10.72 (7.16, 16.05)*
6-Normal RDW, Mod/Severe anemia, Low MCV	7.77 (6.32, 9.55)*	7.99 (6.39, 9.99)*
7-Normal RDW, Mild anemia, High MCV	5.25 (3.42, 8.07)*	3.58 (2.28, 5.62)*
8-Normal RDW, Mild anemia, Low MCV	2.19 (1.65, 2.91)*	2.11 (1.58, 2.82)*
9-High RDW, No anemia, High MCV	11.95 (3.93, 36.36)*	8.44 (2.63, 27.15)*
10-High RDW, No anemia, Low MCV	1.15 (0.74, 1.80)	1.11 (0.70, 1.75)
11-Normal RDW, No anemia, High MCV	2.03 (1.33, 3.10)*	1.74 (1.13, 2.70)*
12-Normal RDW, No anemia, Low MCV	0.99 (0.71, 1.38)	1.08 (0.77, 1.51)
13-High RDW, No anemia, Normal MCV	2.50 (1.78, 3.50)*	1.87 (1.32, 2.65)*
14-High RDW, Mod/Severe anemia, Normal MCV	19.92 (17.50, 22.68)*	14.52 (12.52, 16.84)*
15-High RDW, Mild anemia, Normal MCV	5.56 (4.35, 7.09)*	3.73 (2.88, 4.83)*
16-Normal RDW, Mod/Severe anemia, Normal MCV	13.09 (11.90, 14.40)*	10.83 (9.69, 12.10)*
17-Normal RDW, Mild anemia, Normal MCV	3.45 (3.10, 3.83)*	2.78 (2.49, 3.11)*

*Indicated p < 0.05.

### Nomogram and model performance

**Figure 2:**
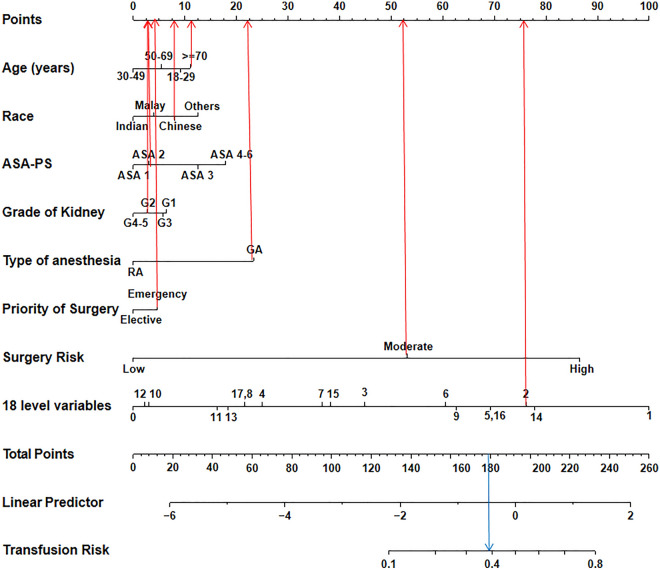
Nomogram estimating transfusion risk in surgery patients.

**Figure 3:**
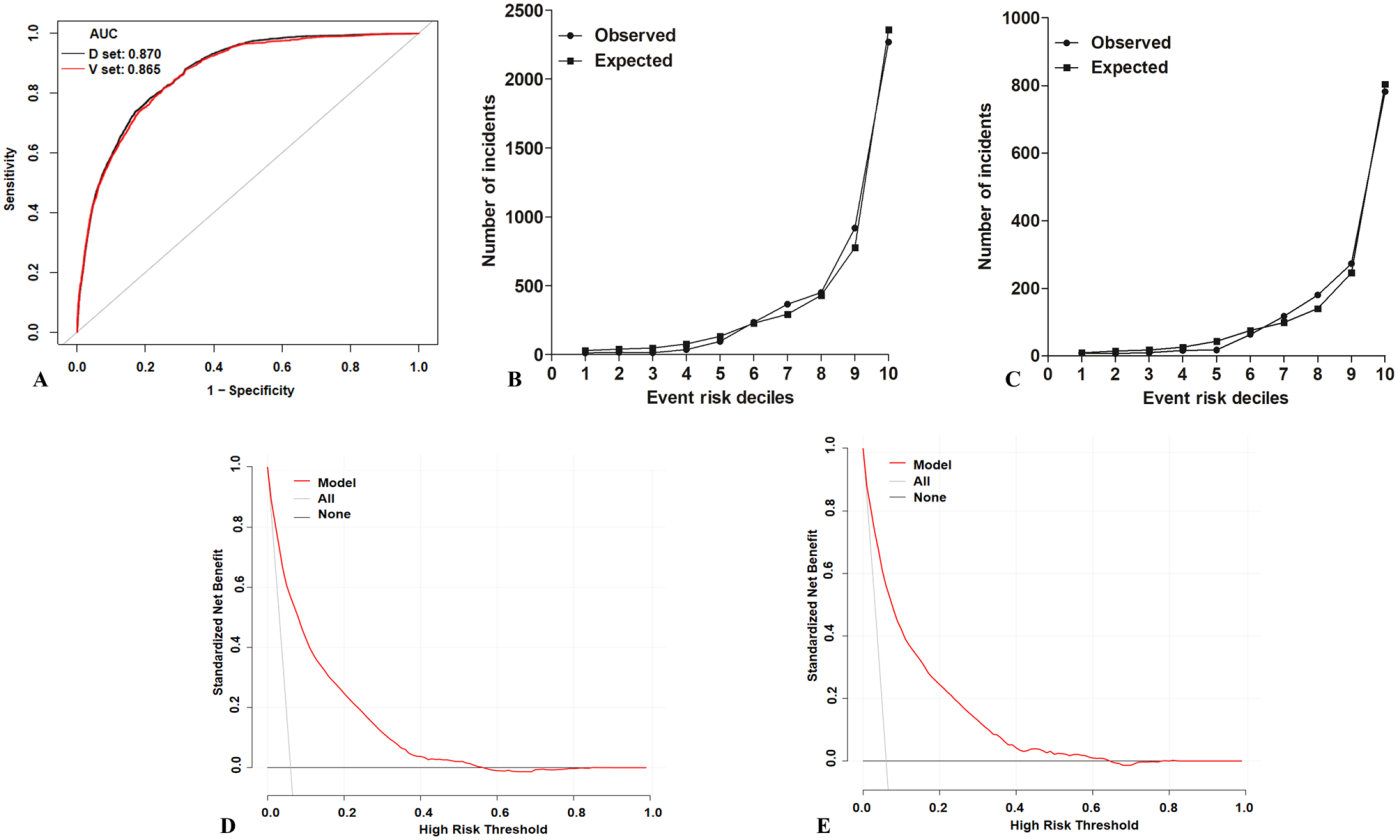
Assessment of a predictive model. (**A**) The receiver operative characteristics curve of the model (D set: derivation cohort; V set: validation cohort). (**B**) The calibration plot of the model in derivation cohort. (**C**) The calibration plot of the model in validation cohort. (**D**) Decision curve analysis for the model in derivation cohort. (**E**) Decision curve analysis for the model in validation cohort.

According to the multivariate logistic regression analysis, eight predictors, including age, race, ASA-PS, grade of kidney disease, type of anesthesia, priority of surgery, surgery risk, and the 18-level variable specified above were included in the model establishment. A nomogram was generated to predict the risk of blood transfusion among patients undergoing surgeries (Fig. 2). The discriminative ability of the prediction model was assessed using the area under the curve (AUC) of the ROC curve (Fig. 3A). The AUC of the derivation cohort was 0.870 (95% CI 0.865–0.875), and the AUC of the validation cohort was 0.865 (95% CI 0.856–0.874), indicating good discriminatory power for the prediction model. The predicted probability (expected) and the actual observation probability (observed) almost coincided in the derivation and validation cohort, indicating consistency between the predicted probability and the actual probability; this result indicates that the model had good calibration (Fig. 3B,C). The calibration plot revealed an adequate model fit for predicting the risk of transfusion.

### The nomogram score system for transfusion risk prediction and clinical utility

After the nomogram is drawn (Fig. 2), each variable corresponds to a point (top line, red arrow). Assigned points for all variables are then summed and can be got total points. Once total points are located, draw vertical line down to bottom line (blue arrow) to obtain predicted probability of risk. For example, patient 1, a woman of Chinese race (8 points) who was aged over 70 years (12 points) and underwent emergency surgery (3 points) with a high RDW, moderate or severe anemia, and a low MCV (level 2: 76 points), a kidney disease grade of G2 (2 points), an ASA-PS of ASA 2 (2 points), GA anesthesia (22 points), and a moderate surgery risk (54 points) received a total score of 179 points (thus showing about 40% predicted risk of blood transfusion). We predicted the presence of transfusion by summing the scores of these eight variables, and the final total scores ranged from 0 to 260. The Youden index identified an optimal cut-off predicted probability of 0.163 with a sensitivity of 0.821 and a specificity of 0.744, and the corresponding total score was about 144. Patients with total scores of ≤ 144 were classified as low risk and patients with total scores of > 144 were classified as high risk^19^. As a result, patient 1 is considered a high risk transfusion population. The decision curve (Fig. 3D,E) shows the clinical value of this model. The patients had a standardized net benefit in the range of a 5–60% likelihood of transfusion risk, meaning that using this model had increased benefits for patients compared to not using this model.

In addition, the model was used to predict transfusion risk in 400 surgical patients from our institution (Supplementary Table 4S). The AUC of ROC was 0.857 (95% CI 0.817–0.897) (Supplementary Fig. 1S) with a total point cut-off value of 140.5 (Supplementary Table 5S).

## Discussion

In this study, we constructed a nomogram model to predict transfusion risk by incorporating the eight independent predictors (age, race, ASA-PS, grade of kidney disease, type of anesthesia, priority of surgery, surgery risk, and the 18-level variable) in surgical patients. The performance of the nomogram was validated internally, and demonstrated good discrimination and calibration, implying that the nomogram had accuracy for predicting transfusion. To confirm the clinical utility, we determined the decisions assisted by this nomogram whether improved patients’ consequences by using DCA. The clinical outcomes based on the threshold probability can be known from this new method. False positive proportion was subtracted from the true positives proportion, and then the relative risk of false positive and false negative results was weighted to obtain the net benefit. It can be gotten from the decision curve that if the threshold probability of a patient was 5–60%, the net benefit was got by using our nomogram to predict transfusion. This nomogram was based on routinely collected preoperative data so as to maximize its clinical applicability, as well as to ensure that it was generalizable and easy to use. According to the weight coefficient score of each predictor in the prediction model, nomogram could be developed into a web-based calculator (http://www.empowerstats.net/pmodel/?m=11633_transfusionpreiction) and linked with electronic medical record systems and/or surgical planning systems. When we input the preoperative information of the surgical patient into this calculator, transfusion probability of patient was obtained. Thereafter, the sensitivity and specificity of this nomogram for estimating the transfusion risk were summarized, and the cut-off value of 0.163 (the corresponding total score was about 144) was identified in the derivation cohort according to the maximum Youden index. Patients with a transfusion probability > 0.163 were considered as high-risk transfusion recipients, and their blood typing and cross-matching should be performed prior to surgery, and PBM should be arranged as soon as possible. Patients with a transfusion probability < 0.163 were considered as low-risk transfusion recipients, they could eliminate the need to complete pretransfusion testing and preoperative blood preparation. In addition, in Wiebe J. de Boer et al.’s study, they found the cut-off value of transfusion risk was 0.168, which was consistent with our findings^20^.

We used this model to predict transfusion risk in surgical patients at our institution and got the cut-off value of total points was 140.5, which was similar to cut-off value of the model (Supplementary Table 5S). Although the transfusion probability in our institutional cohort was higher than in the modeled cohort (Supplementary Table 5S), possibly due to sample size, the cut-off value for dividing high risk patients from low risk were similar between the two cohorts. It suggests that the transfusion predictive model may be suitable for clinical use at our institution. In addition, the conformity of predicted probability (risk) with actual transfusion outcomes in Supplementary Table 4S indicated that 100% of transfused patients and 54.55% of non-transfused patients could benefit from this predictive model at our institution. So, this model might help classify the risk profiles for individual patients undergoing blood transfusion and indicate the necessity of implementing PBM measures as thoroughly as possible in a specific patient^21,22^. The model might also alert surgeons to confirm the possibility of blood transfusions. Ultimately, the decision regarding blood typing, cross-matching, and RBC transfusions are made by surgeons. However, the predicted model-based clinical decision support system can be used as evidence to assist surgeons in making the most effective and informed medical decisions.

Predictors such as age and race were related to transfusion risk. Huang et al.^23^ found that race was an independent risk factor for blood transfusion in abdominoplasty among post-bariatric surgery patients, which was consistent with our results. Likewise, Roubinian et al.^24^ found that gender, race, and age were useful for transfusion prediction modeling with a large-scale dataset including 275,874 inpatients. Chronic kidney disease is related to the occurrence of anemia^25,26^, and may increase the possibility of patients requiring blood transfusion. Our study and a previous study^27^, found that chronic kidney disease grade was meaningful as an independent preoperative blood transfusion risk factor. In contrast to conventional blood transfusion indicators, this study also included surgical strategies, such as the level of anesthesia, type of anaesthesia and surgery priority as modeling variables, which might help supplement various shortcomings to more accurately evaluate the patients’ basic condition^28^. In addition, we incorporated the ASA-PS as an independent transfusion risk predictor in the current prediction model. As mentioned above, the ASA-PS is an anesthesia risk evaluation standard proposed by the American Association of Anesthesiologists. Previous studies have indicated that the ASA-PS could be an independent risk predictor for postoperative death and various complications^10,29^. Hart et al.^30^ found that ASA grade was highly correlated with blood transfusion risk during the perioperative period within hip replacement surgery. High risk surgeries are also associated with high risk blood transfusions^9^. We incorporated surgical risk and the ASA-PS score into the prediction model in order to apply this methodology to the potential need for perioperative transfusions among individual patients and across a wide range of operation types. The outcome variable in this study was receiving > 1 unit red blood cell transfusion perioperatively, which was common in some perioperative transfusion prediction models^31^.

Not surprisingly, 18-level variable was a strong predictor of transfusion in our current model. The AUC of the 18-level variable was 0.762, which was higher than other variables (Supplementary Table 6S). It is known that preoperative anemia is a strong predictor for blood transfusion, and anemia commonly occurs in the population of interest. However, not all surgery patients with preoperative anemia were at risk for RBCs transfusion^20^. It indicated that other predictors might be involved in the stratification of transfusion risk. In our study, the discriminative ability of surgery risk (AUC: 0.741) was similar to that of anemia (AUC: 0.748) (Supplementary Table 6S). Our prediction model includes eight predict factors that could be used for risk stratification. We will focus on early intervention in patients with RBCs transfusion predicted risk > 0.163, rather than all patients with preoperative anemia. Anemia, as defined via hemoglobin concentration, was used as the standard for RBC transfusion^32^ while ignoring the impact of other laboratory test results such as RDW and MCV. RDW is an index of erythrocyte size heterogeneity, MCV measures the average volume of circulatory red blood cells, and abnormal RDW and MCV values are associated with a higher risk of transfusion^33^. Dai et al. found that patients with macrocytic anemia had higher median hemoglobin levels (i.e., were less anemic) than patients with microcytic anemia, yet they were the most likely to receive RBC transfusions^34^. Anemia is one of the most important variables for predicting transfusion risk, but RDW and MCV provide additional prognostic value. All these measures should be considered when estimating transfusion risk in patients undergoing surgery^33^. Beyond hemoglobin level, anemia can be classified via RDW and MCV, and there is an established association between RDW, MCV and transfusion risk. In our study, we included preoperative anemia, MCV, and RDW as predictive factors and found interactions between RDW, MCV, and anemia that were associated with transfusion risk. Many studies have reported interactions between MCV, RDW, and anemia. Anthony et al. showed multiplicative interactions between MCV and RDW in predicting mortality among patients with or without anemia^35^. Kor et al.^36^ reported an interaction between MCV and RDW in predicting mortality within chronic kidney disease. Therefore, we performed further subgroup analyses based on an 18-level variable stratified by RDW, MCV, and anemia categories.

A substantial strength of this research is that our transfusion prediction model can evaluate the efficacy of using a single model to preoperatively predict perioperative transfusion needs for patients over a broad spectrum of operations. Our findings substantially extend prior research, which has applied this methodology to a single type of operation or disease. Moreover, our model showed better discrimination (ROC-AUC: 0.87) for identifying patients at high risk of needing blood transfusions. In addition to these substantial strengths, this study has some limitations. Firstly, although we internally validated our model in order to assess model performance, it is necessary to externally validate the model further by using a multi-centre large samples cohort. Secondly, MCH is possible a better marker of anaemia^37^, But data sources limit access to MCH variables. However, MCH tends to trend with MCV^38^; the application of MCV can make up for this deficiency. And we will investigate the value of MCH in the transfusion risk model in a subsequent study. Thirdly, the outcome of RBCs transfusion within 30 days after surgery may be beyond the period of direct observation/intervention. The benefits of the model might be more temporally focused in the 48–72 h following surgery when pre-operative typing expenditures or blood resource management might be more relevant. Fourthly, we only included preoperative variables in our model, and other factors such as fibrosis severity and blood loss were not included. The accuracy of the model was inevitably affected by these factors. Nevertheless, clinicians might use the predicted results of the current model as a reference for making blood transfusion decisions.

In conclusion, in the current study, we established a new nomogram model predicting patients’ perioperative transfusion needs over a wide spectrum of operations.

## Supplementary Information

Supplementary Figure S1.

Supplementary Information 1.

Supplementary Information 2.

Supplementary Information 3.

Supplementary Information 4.

## Data Availability

The data used for this study, though not available in a public repository, will be made available to other researchers upon reasonable request. Please contact the corresponding authors Ning Li (liningxy@csu.edu.cn) or Run Yao (yaorunxy@csu.edu.cn) via E-mail.
