# Supplementary material for: A novel model forecasting perioperative red blood cell transfusion
Source: Sci Rep. 2022 Sep 27;12:16127. doi: 10.1038/s41598-022-20543-7 (PMC9514715; doi:10.1038/s41598-022-20543-7)
Supplement: Supplementary file 1 — Supplementary Figure S1. [file 41598_2022_20543_MOESM1_ESM.docx]

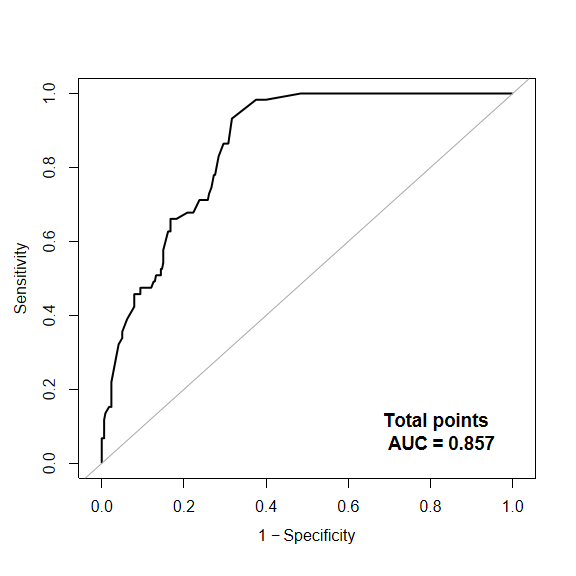


**Supplementary Figure 1S: The receiver operative characteristics curve of the clinic cohort**

Figure legend

Supplementary Figure 1S: The receiver operative characteristics curve of the clinic cohort
