# Supplementary material for: A novel model forecasting perioperative red blood cell transfusion
Source: Sci Rep. 2022 Sep 27;12:16127. doi: 10.1038/s41598-022-20543-7 (PMC9514715; doi:10.1038/s41598-022-20543-7)
Supplement: Supplementary file 2 — Supplementary Information 1. [file 41598_2022_20543_MOESM2_ESM.docx]

**Table 1S Surgery Risk Definitions**

| **Risk grades** | **Definitions** |
| --- | --- |
| Low-Risk Surgery | includes superficial surgery, breast, dental, thyroid, eye, gynaecology, orthopaedics (meniscectomy), urology (transurethral resection, prostate) |
| Moderate-Risk Surgery | intraperitoneal (splenectomy, hiatal hernia repair, cholecystectomy), peripheral artery angioplasty, endovascular aneurysm repair, head and neck surgery, orthopaedics (hip and spine surgery), urology or gynaecology, kidney transplant |
| High-Risk Surgery | surgery includes aortic and great vessel surgery, open lower limb revascularization, amputation, thromboembolectomy, duodenal pancreas surgery, liver resection, bile duct surgery, esophagectomy, repair of intestinal perforation, adrenalectomy, total cystectomy, pneumonectomy, lung, or liver transplantation. |

| **Grades** | **Definitions** |
| --- | --- |
| Ⅰ | healthy body, good development and nutrition, and normal organ function |
| Ⅱ | mild comorbidity except for medical diseases, complete functional compensation |
| Ⅲ | Coexisting illness is serious, physical activity is restricted, but it can still cope with daily activities |
| Ⅳ | Coexisting disease is serious, loss of daily activities, often facing life threat |
| Ⅴ | Near-death patients whose lives cannot be maintained for 24 hours regardless of operation or not |
| Ⅵ | it is confirmed that it is brain death, and its organ is intended to be used for organ transplantation |

**Table2S ASA-PS Classification Definitions**
