# Supplementary material for: A novel model forecasting perioperative red blood cell transfusion
Source: Sci Rep. 2022 Sep 27;12:16127. doi: 10.1038/s41598-022-20543-7 (PMC9514715; doi:10.1038/s41598-022-20543-7)
Supplement: Supplementary file 3 — Supplementary Information 2. [file 41598_2022_20543_MOESM3_ESM.docx]

**Table 3S Descriptive data for study in** **derivation and validation cohort.**

| **Group** | **Derivation cohort**  **(n=73082)** | **Validation cohort**  **(n=24361)** | **P-value** |
| --- | --- | --- | --- |
| **Age (years)** |  |  | **1.000** |
| 18-29 | 8449 (11.56%) | 2814 (11.55%) |  |
| 30-49 | 21081 (28.85%) | 7025 (28.84%) |  |
| 50-69 | 31105 (42.56%) | 10370 (42.57%) |  |
| >= 70 | 12447 (17.03%) | 4152 (17.04%) |  |
| **Gender** |  |  | **0.990** |
| Female | 37973 (51.96%) | 12659 (51.96%) |  |
| Male | 35109 (48.04%) | 11702 (48.04%) |  |
| **Race** |  |  | 0.432 |
| Chinese | 52064 (71.24%) | 17421 (71.51%) |  |
| Malay | 7406 (10.13%) | 2420 (9.93%) |  |
| Indian | 6438 (8.81%) | 2183 (8.96%) |  |
| Others | 7159 (9.80%) | 2335 (9.58%) |  |
| **ASA-PS** |  |  | 1.000 |
| ASA 1 | 16610 (22.73%) | 5537 (22.73%) |  |
| ASA 2 | 37689 (51.57%) | 12563 (51.57%) |  |
| ASA 3 | 13344 (18.26%) | 4449 (18.26%) |  |
| ASA 4-6 | 1613 (2.21%) | 537 (2.20%) |  |
| **Cerebrovascular Accidents** |  |  | 0.166 |
| No | 48742 (66.69%) | 16355 (67.14%) |  |
| Yes | 1388 (1.90%) | 423 (1.74%) |  |
| **Ischemic Heart Disease** |  |  | 0.550 |
| No | 44941 (61.49%) | 15012 (61.62%) |  |
| Yes | 5005 (6.85%) | 1707 (7.01%) |  |
| **Congestive Heart Failure** |  |  | 0.618 |
| No | 50682 (69.35%) | 16937 (69.53%) |  |
| Yes | 1048 (1.43%) | 365 (1.50%) |  |
| **Diabetes Mellitus on insulin** |  |  | 0.144 |
| No | 49420 (67.62%) | 16592 (68.11%) |  |
| Yes | 1781 (2.44%) | 548 (2.25%) |  |
| **Grade of Kidney disease** |  |  | 0.262 |
| G1 | 37777 (51.69%) | 12499 (51.31%) |  |
| G2 | 19186 (26.25%) | 6454 (26.49%) |  |
| G3 | 4418 (6.05%) | 1432 (5.88%) |  |
| G4-5 | 2630 (3.60%) | 847 (3.48%) |  |
| **Type of anesthesia** |  |  | 0.308 |
| General anesthesia | 62276 (85.21%) | 20824 (85.48%) |  |
| Regional/spinal anesthesia | 10806 (14.79%) | 3537 (14.52%) |  |
| **Priority of Surgery** |  |  | 0.125 |
| Elective | 58538 (80.10%) | 19623 (80.55%) |  |
| Emergency | 14544 (19.90%) | 4738 (19.45%) |  |
| **Surgery Risk** |  |  | 0.305 |
| Low | 35986 (49.24%) | 12016 (49.32%) |  |
| Moderate | 29830 (40.82%) | 9894 (40.61%) |  |
| High | 3497 (4.79%) | 1129 (4.63%) |  |
| **18 level variables** |  |  | 0.811 |
| 0-Normal RDW, No anemia, Normal MCV | 44874 (61.40%) | 14964 (61.43%) |  |
| 1-High RDW, Mod/Severe anemia, High MCV | 103 (0.14%) | 32 (0.13%) |  |
| 2-High RDW, Mod/Severe anemia, Low MCV | 2262 (3.10%) | 733 (3.01%) |  |
| 3-High RDW, Mild anemia, High MCV | 27 (0.04%) | 16 (0.07%) |  |
| 4-High RDW, Mild anemia, Low MCV | 950 (1.30%) | 324 (1.33%) |  |
| 5-Normal RDW, Mod/Severe anemia, High MCV | 162 (0.22%) | 46 (0.19%) |  |
| 6-Normal RDW, Mod/Severe anemia, Low MCV | 747 (1.02%) | 273 (1.12%) |  |
| 7-Normal RDW, Mild anemia, High MCV | 215 (0.29%) | 75 (0.31%) |  |
| 8-Normal RDW, Mild anemia, Low MCV | 1065 (1.46%) | 337 (1.38%) |  |
| 9-High RDW, No anemia, High MCV | 18 (0.02%) | 7 (0.03%) |  |
| 10-High RDW, No anemia, Low MCV | 746 (1.02%) | 251 (1.03%) |  |
| 11-Normal RDW, No anemia, High MCV | 497 (0.68%) | 171 (0.70%) |  |
| 12-Normal RDW, No anemia, Low MCV | 1599 (2.19%) | 541 (2.22%) |  |
| 13-High RDW, No anemia, Normal MCV | 657 (0.90%) | 206 (0.85%) |  |
| 14-High RDW, Mod/Severe anemia, Normal MCV | 1348 (1.84%) | 443 (1.82%) |  |
| 15-High RDW, Mild anemia, Normal MCV | 665 (0.91%) | 206 (0.85%) |  |
| 16-Normal RDW, Mod/Severe anemia, Normal MCV | 3918 (5.36%) | 1354 (5.56%) |  |
| 17-Normal RDW, Mild anemia, Normal MCV | 7443 (10.18%) | 2414 (9.91%) |  |
| **Transfusion** |  |  | 1.000 |
| No | 68666 (93.96%) | 22889 (93.96%) |  |
| Yes | 4416 (6.04%) | 1472 (6.04%) |  |
