# Supplementary material for: A novel model forecasting perioperative red blood cell transfusion
Source: Sci Rep. 2022 Sep 27;12:16127. doi: 10.1038/s41598-022-20543-7 (PMC9514715; doi:10.1038/s41598-022-20543-7)
Supplement: Supplementary file 4 — Supplementary Information 3. [file 41598_2022_20543_MOESM4_ESM.docx]

**Table4S Application of this model to predict the transfusion probability of 400 surgical patients in our institution.**

| Patient | Diagnosis | Age | Race | GKD | Type of  anesthesia | Priority of  Surgery | Surgery  Risk | 18 level  variables | ASA  -PS | Total  points | Predict  Transfusion risk | Predict  Transfusion  outcome | Transfusion outcome |
| --- | --- | --- | --- | --- | --- | --- | --- | --- | --- | --- | --- | --- | --- |
| 2 | Sigmoid Colon Malignant Tumor | 86(12) | Chinese(8) | G4(0) | GA(22) | Elective(0) | High(85) | 14(78) | Ⅳ(18) | 223 | 0.777(High) | yes | yes |
| 3 | Gastrointestinal anastomotic ulcer with bleeding | 62(5) | Chinese(8) | G1(8) | GA(22) | Elective(0) | High(85) | 16(70) | Ⅲ(12) | 210 | 0.702(High) | yes | no |
| 4 | Gastrointestinal bleeding | 71(12) | Chinese(8) | G4(0) | GA(22) | Elective(0) | High(85) | 16(70) | Ⅳ(18) | 215 | 0.803(High) | yes | yes |
| 5 | Placenta | 37(0) | Chinese(8) | G1(8) | GA(22) | Elective(0) | High(85) | 3(45) | Ⅲ(12) | 180 | 0.423(High) | yes | no |
| 6 | Intracranial space-occupying lesions | 48(0) | Chinese(8) | G2(2) | GA(22) | Elective(0) | High(85) | 0(0) | Ⅱ(2) | 119 | 0.091(Low) | no | no |
| 7 | Skull base space occupying lesions | 35(0) | Chinese(8) | G2(2) | GA(22) | Elective(0) | High(85) | 0(0) | Ⅱ(2) | 119 | 0.091(Low) | no | no |
| 8 | Pulmonary nodules | 64(5) | Chinese(8) | G2(2) | GA(22) | Elective(0) | High(85) | 5(70) | Ⅱ(2) | 194 | 0.53(High) | yes | no |
| 9 | Hepatic space-occupying lesions | 38(0) | Chinese(8) | G2(2) | GA(22) | Elective(0) | High(85) | 17(20) | Ⅱ(2) | 139 | 0.199(High) | yes | no |
| 10 | Stomach cancer | 71(12) | Chinese(8) | G1(8) | GA(22) | Elective(0) | High(85) | 14(78) | Ⅲ(12) | 225 | 0.786(High) | yes | yes |
| 11 | Uterine mass | 36(0) | Chinese(8) | G2(2) | GA(22) | Elective(0) | High(85) | 17(20) | Ⅱ(2) | 139 | 0.199(High) | yes | no |
| 12 | Pelvic mass | 31(0) | Chinese(8) | G1(8) | GA(22) | Elective(0) | High(85) | 2(76) | Ⅱ(2) | 201 | 0.533(High) | yes | no |
| 13 | Aseptic necrosis of femoral head | 81(12) | Chinese(8) | G2(2) | GA(22) | Elective(0) | High(85) | 0(0) | Ⅲ(12) | 141 | 0.231(High) | yes | no |
| 14 | Mediastinal mass | 64(5) | Chinese(8) | G2(2) | GA(22) | Elective(0) | High(85) | 0(0) | Ⅱ(2) | 124 | 0.117(Low) | no | no |
| 15 | Intraspinal mass | 54(5) | Chinese(8) | G2(2) | GA(22) | Elective(0) | High(85) | 16(70) | Ⅱ(2) | 194 | 0.552(High) | yes | no |
| 16 | Rectal cancer | 54(5) | Chinese(8) | G2(2) | GA(22) | Elective(0) | High(85) | 13(18) | Ⅱ(2) | 142 | 0.194(High) | yes | no |
| 17 | Bladder malignant tumor | 72(12) | Chinese(8) | G3(7) | GA(22) | Elective(0) | Moderate(52) | 16(70) | Ⅲ(12) | 183 | 0.487(High) | yes | no |
| 18 | Cervical spinal stenosis | 59(5) | Chinese(8) | G3(7) | GA(22) | Elective(0) | High(85) | 0(0) | Ⅱ(2) | 129 | 0.117(Low) | no | no |
| 19 | Small bowel tumors | 66(5) | Chinese(8) | G2(2) | GA(22) | Elective(0) | Moderate(52) | 0(0) | Ⅲ(12) | 101 | 0.08(Low) | no | no |
| 20 | Acute appendicitis | 77(12) | Chinese(8) | G2(2) | GA(22) | Emergency(3) | Moderate(52) | 0(0) | Ⅲ(12) | 111 | 0.094(Low) | no | no |
| 21 | Femoral osteomyelitis | 60(5) | Chinese(8) | G2(2) | GA(22) | Elective(0) | High(85) | 14(78) | Ⅲ(12) | 212 | 0.757(High) | yes | yes |
| 43 | Colon cancer | 63(5) | Chinese(8) | G2(2) | GA(22) | Elective(0) | High(85) | 2(76) | Ⅳ(18) | 216 | 0.809(High) | yes | yes |
| 85 | Bladder Cancer | 84(12) | Chinese(8) | G2(2) | GA(22) | Elective(0) | High(85) | 2(76) | Ⅳ(18) | 223 | 0.833(High) | yes | yes |
| 59 | Uterine fibroids | 33(0) | Chinese(8) | G2(2) | GA(22) | Elective(0) | High(85) | 2(76) | Ⅱ(2) | 195 | 0.533(High) | yes | no |
| 74 | Serous cystadenoma | 45(0) | Chinese(8) | G2(2) | GA(22) | Elective(0) | High(85) | 2(76) | Ⅲ(12) | 205 | 0.686(High) | yes | no |
| 34 | Chronic left heart failure | 48(0) | Chinese(8) | G3(7) | GA(22) | Elective(0) | High(85) | 2(76) | Ⅳ(18) | 216 | 0.762(High) | yes | yes |
| 66 | Liver cancer | 51(5) | Chinese(8) | G2(2) | GA(22) | Elective(0) | High(85) | 2(76) | Ⅲ(12) | 210 | 0.743(High) | yes | yes |
| 87 | Uterine fibroids | 49(0) | Chinese(8) | G1(8) | GA(22) | Elective(0) | High(85) | 6(60) | Ⅱ(2) | 185 | 0.423(High) | yes | no |
| 64 | Leukoplakia | 45(0) | Chinese(8) | G2(2) | GA(22) | Elective(0) | High(85) | 10(3) | Ⅱ(2) | 122 | 0.109(Low) | no | no |
| 96 | Uterine neoplasms | 31(0) | Chinese(8) | G1(8) | GA(22) | Elective(0) | High(85) | 10(3) | Ⅱ(2) | 128 | 0.109(Low) | no | no |
| 53 | Kidney Cancer | 28(10) | Chinese(8) | G1(8) | GA(22) | Elective(0) | High(85) | 10(3) | Ⅲ(12) | 148 | 0.245(High) | yes | no |
| 78 | Scoliosis | 19(10) | Chinese(8) | G1(8) | GA(22) | Elective(0) | High(85) | 15(40) | Ⅲ(12) | 185 | 0.45(High) | yes | yes |
| 97 | Congenital heart disease | 44(0) | Chinese(8) | G2(2) | GA(22) | Elective(0) | High(85) | 15(40) | Ⅲ(12) | 169 | 0.371(High) | yes | no |
| 33 | Threatened abortion | 35(0) | Chinese(8) | G1(8) | GA(22) | Emergency(3) | Moderate(52) | 15(40) | Ⅲ(12) | 145 | 0.169(High) | yes | yes |
| 49 | Kidney atrophy | 36(0) | Chinese(8) | G2(2) | GA(22) | Elective(0) | High(85) | 17(20) | Ⅲ(12) | 149 | 0.323(High) | yes | yes |
| 88 | Ureteral stricture | 73(12) | Chinese(8) | G3(7) | GA(22) | Elective(0) | High(85) | 17(20) | Ⅱ(2) | 156 | 0.279(High) | yes | no |
| 62 | Colon cancer | 57(5) | Chinese(8) | G2(2) | GA(22) | Elective(0) | High(85) | 17(20) | Ⅲ(12) | 154 | 0.387(High) | yes | no |
| 27 | Craniopharyngioma | 32(0) | Chinese(8) | G1(8) | GA(22) | Elective(0) | High(85) | 17(20) | Ⅲ(12) | 155 | 0.323(High) | yes | yes |
| 48 | Aortic dissection | 49(0) | Chinese(8) | G3(7) | GA(22) | Emergency(3) | High(85) | 14(78) | Ⅳ(18) | 221 | 0.777(High) | yes | yes |
| 35 | Stomach cancer | 70(12) | Chinese(8) | G1(8) | GA(22) | Elective(0) | High(85) | 14(78) | Ⅳ(18) | 231 | 0.843(High) | yes | yes |
| 39 | Right kidney occupancy | 71(12) | Chinese(8) | G4(0) | GA(22) | Elective(0) | High(85) | 14(78) | Ⅲ(12) | 217 | 0.786(High) | yes | yes |
| 40 | Colon cancer | 59(5) | Chinese(8) | G1(8) | GA(22) | Elective(0) | High(85) | 16(70) | Ⅳ(18) | 216 | 0.776(High) | yes | yes |
| 77 | Endocarditis | 39(0) | Chinese(8) | G1(8) | GA(22) | Elective(0) | High(85) | 16(70) | Ⅲ(12) | 205 | 0.641(High) | yes | no |
| 56 | Ovarian cancer | 44(0) | Chinese(8) | G1(8) | GA(22) | Elective(0) | High(85) | 16(70) | Ⅳ(18) | 211 | 0.724(High) | yes | yes |
| 55 | Ovarian tumors | 67(5) | Chinese(8) | G2(2) | GA(22) | Elective(0) | Moderate(52) | 16(70) | Ⅱ(2) | 161 | 0.295(High) | yes | no |
| 38 | Benign prostatic hyperplasia | 58(5) | Chinese(8) | G2(2) | GA(22) | Elective(0) | Moderate(52) | 16(70) | Ⅱ(2) | 161 | 0.295(High) | yes | no |
| 30 | Ascending colon tumor | 60(5) | Chinese(8) | G4(0) | GA(22) | Elective(0) | High(85) | 16(70) | Ⅳ(18) | 208 | 0.776(High) | yes | yes |
| 80 | Lumbar spinal stenosis | 72(12) | Chinese(8) | G2(2) | GA(22) | Elective(0) | High(85) | 16(70) | Ⅲ(12) | 211 | 0.735(High) | yes | yes |
| 73 | Brain herniation | 36(0) | Chinese(8) | G2(2) | GA(22) | Emergency(3) | High(85) | 16(70) | Ⅳ(18) | 208 | 0.725(High) | yes | yes |
| 103 | Spondylolisthesis | 70(12) | Chinese(8) | G2(2) | GA(22) | Elective(0) | High(85) | 16(70) | Ⅳ(18) | 217 | 0.803(High) | yes | yes |
| 29 | Spinal tuberculosis | 61(5) | Chinese(8) | G2(2) | GA(22) | Elective(0) | High(85) | 16(70) | Ⅲ(12) | 204 | 0.702(High) | yes | yes |
| 36 | Hydronephrosis | 51(5) | Chinese(8) | G2(2) | GA(22) | Elective(0) | High(85) | 13(18) | Ⅲ(12) | 152 | 0.315(High) | yes | yes |
| 70 | Gallstones | 69(5) | Chinese(8) | G2(2) | GA(22) | Elective(0) | High(85) | 13(18) | Ⅱ(2) | 142 | 0.194(High) | yes | no |
| 102 | Meningioma | 48(0) | Chinese(8) | G2(2) | GA(22) | Elective(0) | High(85) | 0(0) | Ⅳ(18) | 135 | 0.221(High) | yes | yes |
| 45 | Cerebral hemorrhage | 33(0) | Chinese(8) | G1(8) | GA(22) | Emergency(3) | High(85) | 0(0) | Ⅳ(18) | 144 | 0.222(High) | yes | no |
| 71 | Kidney Cancer | 45(0) | Chinese(8) | G2(2) | GA(22) | Elective(0) | High(85) | 0(0) | Ⅲ(12) | 132 | 0.162(Low) | no | no |
| 41 | Vaginal polyps | 56(5) | Chinese(8) | G2(2) | GA(22) | Elective(0) | High(85) | 0(0) | Ⅱ(2) | 124 | 0.117(Low) | no | no |
| 44 | Cesarean section | 30(0) | Chinese(8) | G2(2) | RA(0) | Elective(0) | Moderate(52) | 0(0) | Ⅱ(2) | 64 | 0.014(Low) | no | no |
| 42 | Prostate cancer | 50(5) | Chinese(8) | G2(2) | GA(22) | Elective(0) | High(85) | 0(0) | Ⅲ(12) | 134 | 0.203(High) | yes | no |
| 72 | Spinal cord degeneration | 58(5) | Chinese(8) | G2(2) | GA(22) | Elective(0) | High(85) | 0(0) | Ⅲ(12) | 134 | 0.203(High) | yes | no |
| 86 | Cerebral aneurysm | 52(5) | Chinese(8) | G2(2) | GA(22) | Elective(0) | High(85) | 0(0) | Ⅲ(12) | 134 | 0.203(High) | yes | no |
| 63 | Colon cancer | 64(5) | Chinese(8) | G2(2) | GA(22) | Elective(0) | High(85) | 0(0) | Ⅲ(12) | 134 | 0.203(High) | yes | no |
| 32 | Frontal parietal lesions | 28(10) | Chinese(8) | G2(2) | GA(22) | Elective(0) | High(85) | 0(0) | Ⅳ(18) | 145 | 0.282(High) | yes | yes |
| 93 | Cervical cancer | 49(0) | Chinese(8) | G1(8) | GA(22) | Elective(0) | Moderate(52) | 0(0) | Ⅲ(12) | 102 | 0.062(Low) | no | no |
| 76 | Dysfetal neuroepithelioma | 57(5) | Chinese(8) | G2(2) | GA(22) | Elective(0) | High(85) | 0(0) | Ⅲ(12) | 134 | 0.203(High) | yes | no |
| 91 | Ureteral stricture | 56(5) | Chinese(8) | G2(2) | GA(22) | Elective(0) | Low(0) | 0(0) | Ⅱ(2) | 39 | 0.011(Low) | no | no |
| 58 | Epidermoid cyst | 30(0) | Chinese(8) | G1(8) | GA(22) | Elective(0) | High(85) | 0(0) | Ⅱ(2) | 125 | 0.091(Low) | no | no |
| 52 | Right cheek cancer | 47(0) | Chinese(8) | G2(2) | GA(22) | Elective(0) | High(85) | 0(0) | Ⅲ(12) | 129 | 0.162(Low) | no | no |
| 54 | Cheek cancer | 45(0) | Chinese(8) | G1(8) | GA(22) | Elective(0) | High(85) | 0(0) | Ⅲ(12) | 135 | 0.162(Low) | no | no |
| 67 | Tongue cancer | 62(5) | Chinese(8) | G1(8) | GA(22) | Elective(0) | High(85) | 0(0) | Ⅳ(18) | 146 | 0.272(High) | yes | yes |
| 100 | Malignant tumor of palate | 61(5) | Chinese(8) | G2(2) | GA(22) | Elective(0) | High(85) | 0(0) | Ⅲ(12) | 134 | 0.203(High) | yes | no |
| 90 | Kidney tumors | 46(0) | Chinese(8) | G2(2) | GA(22) | Elective(0) | High(85) | 0(0) | Ⅲ(12) | 129 | 0.162(Low) | no | no |
| 37 | Uterine prolapse | 62(5) | Chinese(8) | G2(2) | GA(22) | Elective(0) | Moderate(52) | 0(0) | Ⅱ(2) | 91 | 0.043(Low) | no | no |
| 99 | Sellar mass | 53(5) | Chinese(8) | G2(2) | GA(22) | Elective(0) | High(85) | 0(0) | Ⅲ(12) | 134 | 0.203(High) | yes | no |
| 47 | Lumbar spinal stenosis | 21(10) | Chinese(8) | G1(8) | GA(22) | Elective(0) | High(85) | 0(0) | Ⅲ(12) | 145 | 0.211(High) | yes | no |
| 89 | Kidney stones | 65(5) | Chinese(8) | G2(2) | GA(22) | Elective(0) | Moderate(52) | 0(0) | Ⅱ(2) | 91 | 0.043(Low) | no | no |
| 94 | Lung cancer | 83(12) | Chinese(8) | G2(2) | GA(22) | Elective(0) | High(85) | 0(0) | Ⅲ(12) | 141 | 0.231(High) | yes | no |
| 84 | Intracranial space-occupying lesions | 42(0) | Chinese(8) | G2(2) | GA(22) | Elective(0) | High(85) | 0(0) | Ⅲ(12) | 129 | 0.162(Low) | no | no |
| 82 | Congenital heart disease | 43(0) | Chinese(8) | G2(2) | GA(22) | Elective(0) | High(85) | 0(0) | Ⅲ(12) | 129 | 0.162(Low) | no | no |
| 46 | Valvular heart disease | 67(5) | Chinese(8) | G3(7) | GA(22) | Elective(0) | High(85) | 0(0) | Ⅲ(12) | 139 | 0.203(High) | yes | no |
| 60 | Chiaris deformity | 57(5) | Chinese(8) | G1(8) | GA(22) | Elective(0) | High(85) | 0(0) | Ⅲ(12) | 140 | 0.203(High) | yes | no |
| 81 | Lumbar spinal stenosis | 25(10) | Chinese(8) | G1(8) | GA(22) | Elective(0) | High(85) | 0(0) | Ⅲ(12) | 145 | 0.211(High) | yes | no |
| 61 | Glioma | 36(0) | Chinese(8) | G2(2) | GA(22) | Elective(0) | High(85) | 0(0) | Ⅲ(12) | 129 | 0.162(Low) | no | no |
| 83 | Intracranial space-occupying lesions | 50(5) | Chinese(8) | G2(2) | GA(22) | Elective(0) | High(85) | 0(0) | Ⅲ(12) | 134 | 0.203(High) | yes | no |
| 75 | Lumbar disc herniation | 50(5) | Chinese(8) | G2(2) | GA(22) | Elective(0) | High(85) | 0(0) | Ⅲ(12) | 134 | 0.203(High) | yes | no |
| 68 | Drug-induced femoral head necrosis | 29(10) | Chinese(8) | G1(8) | RA(0) | Elective(0) | High(85) | 0(0) | Ⅲ(12) | 123 | 0.097(Low) | no | no |
| 51 | Tongue cancer | 59(5) | Chinese(8) | G2(2) | GA(22) | Elective(0) | High(85) | 0(0) | Ⅲ(12) | 134 | 0.203(High) | yes | no |
| 95 | Cesarean section | 52(5) | Chinese(8) | G1(8) | RA(0) | Elective(0) | Moderate(52) | 0(0) | Ⅲ(12) | 85 | 0.034(Low) | no | no |
| 79 | Saddle area occupancy | 62(5) | Chinese(8) | G2(2) | GA(22) | Elective(0) | High(85) | 0(0) | Ⅳ(18) | 140 | 0.272(High) | yes | yes |
| 57 | Endometrial cancer | 49(0) | Chinese(8) | G2(2) | GA(22) | Elective(0) | High(85) | 0(0) | Ⅲ(12) | 129 | 0.162(Low) | no | no |
| 50 | Tongue cancer | 35(0) | Chinese(8) | G2(2) | GA(22) | Elective(0) | High(85) | 0(0) | Ⅳ(18) | 135 | 0.221(High) | yes | no |
| 92 | Pharyngeal lateral wall tumor | 48(0) | Chinese(8) | G1(8) | GA(22) | Elective(0) | High(85) | 0(0) | Ⅲ(12) | 135 | 0.162(Low) | no | no |
| 69 | Pituitary tumor | 52(5) | Chinese(8) | G2(2) | GA(22) | Elective(0) | High(85) | 0(0) | Ⅳ(18) | 140 | 0.272(High) | yes | yes |
| 98 | Pancreatic mass | 37(0) | Chinese(8) | G1(8) | GA(22) | Elective(0) | High(85) | 0(0) | Ⅲ(12) | 135 | 0.162(Low) | no | no |
| 28 | Meningioma | 40(0) | Chinese(8) | G1(8) | GA(22) | Elective(0) | High(85) | 0(0) | Ⅳ(18) | 141 | 0.221(High) | yes | yes |
| 65 | Benign tumor of nasal sulcus | 67(5) | Chinese(8) | G2(2) | GA(22) | Elective(0) | High(85) | 0(0) | Ⅱ(2) | 124 | 0.117(Low) | no | no |
| 101 | Rectal cancer | 67(5) | Chinese(8) | G2(2) | GA(22) | Elective(0) | High(85) | 0(0) | Ⅲ(12) | 134 | 0.203(High) | yes | no |
| 105 | Aortic dissection | 40(0) | Chinese(8) | G1(8) | GA(22) | Emergency(3) | High(85) | 17(20) | Ⅳ(18) | 164 | 0.413(High) | yes | yes |
| 106 | aortic dissection | 55(5) | Chinese(8) | G3(7) | GA(22) | Emergency(3) | High(85) | 0(0) | Ⅳ(18) | 148 | 0.274(High) | yes | yes |
| 109 | aortic dissection | 48(0) | Chinese(8) | G3(7) | GA(22) | Emergency(3) | High(85) | 2(76) | Ⅲ(12) | 213 | 0.688(High) | yes | yes |
| 110 | craniocerebral trauma | 79(12) | Chinese(8) | G3(7) | GA(22) | Elective(0) | High(85) | 1(100) | Ⅳ(18) | 252 | 0.904(High) | yes | yes |
| 111 | loop ileostomy | 66(5) | Chinese(8) | G2(2) | GA(22) | Elective(0) | High(85) | 2(76) | Ⅲ(12) | 210 | 0.743(High) | yes | yes |
| 113 | preeclampsia | 29(10) | Chinese(8) | G1(8) | RA(0) | Elective(0) | High(85) | 0(0) | Ⅱ(2) | 113 | 0.053(Low) | no | no |
| 114 | cervical cancer | 51(5) | Chinese(8) | G1(8) | GA(22) | Elective(0) | High(85) | 0(0) | Ⅲ(12) | 140 | 0.203(High) | yes | no |
| 115 | cesarean delivery | 31(0) | Chinese(8) | G1(8) | RA(0) | Elective(0) | Moderate(52) | 0(0) | Ⅱ(2) | 70 | 0.014(Low) | no | no |
| 116 | ovarian cancer | 58(5) | Chinese(8) | G1(8) | GA(22) | Elective(0) | High(85) | 0(0) | Ⅲ(12) | 140 | 0.203(High) | yes | no |
| 117 | Intrahepatic cholangiolithiasis with cholecystitis | 54(5) | Chinese(8) | G1(8) | GA(22) | Elective(0) | High(85) | 0(0) | Ⅲ(12) | 140 | 0.203(High) | yes | no |
| 118 | thyroid cancer | 38(0) | Chinese(8) | G2(2) | GA(22) | Elective(0) | High(85) | 0(0) | Ⅱ(2) | 119 | 0.091(Low) | no | no |
| 119 | pelvic mass | 82(12) | Chinese(8) | G1(8) | GA(22) | Elective(0) | Moderate(52) | 0(0) | Ⅲ(12) | 114 | 0.093(Low) | no | no |
| 120 | skull defects | 56(5) | Chinese(8) | G1(8) | GA(22) | Elective(0) | Moderate(52) | 16(70) | Ⅳ(18) | 183 | 0.541(High) | yes | yes |
| 121 | fetal distress | 31(0) | Chinese(8) | G2(2) | RA(0) | Elective(0) | High(85) | 0(0) | Ⅲ(12) | 107 | 0.072(Low) | no | no |
| 122 | tubal pregnancy | 32(0) | Chinese(8) | G1(8) | GA(22) | Emergency(3) | Moderate(52) | 17(20) | Ⅲ(12) | 125 | 0.141(Low) | no | no |
| 124 | spinal cord injury | 51(5) | Chinese(8) | G1(8) | GA(22) | Elective(0) | High(85) | 0(0) | Ⅱ(2) | 130 | 0.117(Low) | no | no |
| 125 | spinal stenosis | 58(5) | Chinese(8) | G1(8) | GA(22) | Elective(0) | High(85) | 17(20) | Ⅲ(12) | 160 | 0.387(High) | yes | no |
| 126 | multiple trauma | 66(5) | Chinese(8) | G1(8) | GA(22) | Elective(0) | High(85) | 16(70) | Ⅳ(18) | 216 | 0.776(High) | yes | no |
| 127 | Ileocecal mass | 51(5) | Chinese(8) | G1(8) | GA(22) | Elective(0) | High(85) | 17(20) | Ⅲ(12) | 160 | 0.387(High) | yes | no |
| 128 | cervical internal disc herniation | 59(5) | Chinese(8) | G1(8) | GA(22) | Elective(0) | High(85) | 0(0) | Ⅱ(2) | 130 | 0.117(Low) | no | no |
| 129 | cholecystitis | 49(0) | Chinese(8) | G1(8) | GA(22) | Elective(0) | High(85) | 0(0) | Ⅲ(12) | 135 | 0.162(Low) | no | no |
| 130 | spinal stenosis | 40(0) | Chinese(8) | G1(8) | GA(22) | Elective(0) | High(85) | 0(0) | Ⅲ(12) | 135 | 0.162(Low) | no | no |
| 131 | Fractrue of the upper limb | 20(10) | Chinese(8) | G1(8) | GA(22) | Elective(0) | High(85) | 0(0) | Ⅱ(2) | 135 | 0.123(Low) | no | no |
| 132 | cerebellar hemorrhage | 30(0) | Chinese(8) | G1(8) | GA(22) | Elective(0) | High(85) | 17(20) | Ⅲ(12) | 155 | 0.323(High) | yes | yes |
| 133 | colon carcinoma | 65(5) | Chinese(8) | G2(2) | GA(22) | Elective(0) | High(85) | 16(70) | Ⅳ(18) | 210 | 0.776(High) | yes | no |
| 134 | Abdominal aortic dissection | 49(0) | Chinese(8) | G2(2) | GA(22) | Elective(0) | High(85) | 0(0) | Ⅱ(2) | 119 | 0.091(Low) | no | no |
| 135 | sigmoid colon cancer | 59(5) | Chinese(8) | G2(2) | GA(22) | Elective(0) | High(85) | 0(0) | Ⅲ(12) | 134 | 0.203(High) | yes | no |
| 136 | obstructive jaundice | 52(5) | Chinese(8) | G2(2) | RA(0) | Elective(0) | High(85) | 17(20) | Ⅲ(12) | 132 | 0.201(High) | yes | no |
| 137 | Glioma | 56(5) | Chinese(8) | G2(2) | GA(22) | Elective(0) | High(85) | 0(0) | Ⅱ(2) | 124 | 0.117(Low) | no | no |
| 138 | lumbar intervertebral disc hernia | 39(0) | Chinese(8) | G1(8) | GA(22) | Elective(0) | High(85) | 0(0) | Ⅱ(2) | 125 | 0.091(Low) | no | no |
| 139 | lumbar intervertebral disc hernia | 29(10) | Chinese(8) | G1(8) | RA(0) | Elective(0) | High(85) | 0(0) | Ⅱ(2) | 113 | 0.053(Low) | no | no |
| 140 | spinal cord injury | 68(5) | Chinese(8) | G2(2) | GA(22) | Elective(0) | High(85) | 17(20) | Ⅱ(2) | 144 | 0.247(High) | yes | no |
| 141 | Patellar fracture | 75(12) | Chinese(8) | G2(2) | RA(0) | Elective(0) | High(85) | 0(0) | Ⅲ(12) | 119 | 0.107(Low) | no | no |
| 142 | Jaw malignant tumor | 72(12) | Chinese(8) | G2(2) | GA(22) | Elective(0) | Low(0) | 17(20) | Ⅲ(12) | 76 | 0.06(Low) | no | no |
| 143 | mandibular protrusion | 18(10) | Chinese(8) | G1(8) | GA(22) | Elective(0) | High(85) | 0(0) | Ⅱ(2) | 135 | 0.123(Low) | no | no |
| 144 | Tongue malignant tumor | 36(0) | Chinese(8) | G1(8) | GA(22) | Elective(0) | High(85) | 0(0) | Ⅱ(2) | 125 | 0.091(Low) | no | no |
| 145 | mass in cheek | 51(5) | Chinese(8) | G1(8) | GA(22) | Elective(0) | Low(0) | 0(0) | Ⅱ(2) | 45 | 0.011(Low) | no | no |
| 146 | colon carcinoma | 59(5) | Chinese(8) | G1(8) | GA(22) | Elective(0) | High(85) | 2(76) | Ⅳ(18) | 222 | 0.809(High) | yes | yes |
| 147 | Buccal carcinoma | 64(5) | Chinese(8) | G1(8) | GA(22) | Elective(0) | High(85) | 7(38) | Ⅳ(18) | 184 | 0.528(High) | yes | no |
| 148 | gastroesophageal reflux | 55(5) | Chinese(8) | G1(8) | GA(22) | Elective(0) | Moderate(52) | 0(0) | Ⅱ(2) | 97 | 0.043(Low) | no | no |
| 149 | rectal polyp | 49(0) | Chinese(8) | G1(8) | GA(22) | Elective(0) | Moderate(52) | 0(0) | Ⅱ(2) | 92 | 0.033(Low) | no | no |
| 150 | gastric cancer | 30(0) | Chinese(8) | G1(8) | GA(22) | Elective(0) | High(85) | 0(0) | Ⅱ(2) | 125 | 0.091(Low) | no | no |
| 152 | umbilical cord entangle neck | 25(10) | Chinese(8) | G1(8) | RA(0) | Elective(0) | Moderate(52) | 17(20) | Ⅲ(12) | 110 | 0.082(Low) | no | no |
| 153 | placenta previa | 33(0) | Chinese(8) | G1(8) | GA(22) | Elective(0) | High(85) | 16(70) | Ⅳ(18) | 211 | 0.724(High) | yes | no |
| 154 | perimary hepatic carcinoma | 46(0) | Chinese(8) | G1(8) | RA(0) | Elective(0) | Moderate(52) | 0(0) | Ⅱ(2) | 70 | 0.014(Low) | no | no |
| 155 | coronary heart disease | 66(5) | Chinese(8) | G2(2) | RA(0) | Elective(0) | High(85) | 0(0) | Ⅱ(2) | 102 | 0.05(Low) | no | no |
| 156 | colon carcinoma | 73(12) | Chinese(8) | G3(7) | GA(22) | Elective(0) | High(85) | 14(78) | Ⅳ(18) | 230 | 0.843(High) | yes | yes |
| 157 | elderly primipara | 39(0) | Chinese(8) | G1(8) | RA(0) | Elective(0) | Moderate(52) | 0(0) | Ⅲ(12) | 80 | 0.026(Low) | no | no |
| 158 | Ovarian teratoma | 23(10) | Chinese(8) | G1(8) | GA(22) | Elective(0) | High(85) | 0(0) | Ⅱ(2) | 135 | 0.123(Low) | no | no |
| 159 | Liver failure | 49(0) | Chinese(8) | G3(7) | GA(22) | Elective(0) | High(85) | 2(76) | Ⅳ(18) | 216 | 0.762(High) | yes | yes |
| 160 | intrahepatic cholestasis of pregnancy | 36(0) | Chinese(8) | G1(8) | RA(0) | Elective(0) | High(85) | 17(20) | Ⅲ(12) | 133 | 0.16(Low) | no | no |
| 161 | cervical cancer | 46(0) | Chinese(8) | G1(8) | GA(22) | Elective(0) | High(85) | 0(0) | Ⅳ(18) | 141 | 0.221(High) | yes | yes |
| 162 | scar uterus | 33(0) | Chinese(8) | G1(8) | GA(22) | Elective(0) | High(85) | 1(100) | Ⅳ(18) | 241 | 0.858(High) | yes | yes |
| 163 | aortic dissection | 37(0) | Chinese(8) | G2(2) | GA(22) | Emergency(3) | High(85) | 0(0) | Ⅳ(18) | 138 | 0.222(High) | yes | no |
| 164 | endometrial adenocarcinoma | 51(5) | Chinese(8) | G2(2) | GA(22) | Elective(0) | High(85) | 0(0) | Ⅱ(2) | 124 | 0.117(Low) | no | no |
| 165 | intrauterine adhesion | 35(0) | Chinese(8) | G1(8) | GA(22) | Elective(0) | High(85) | 0(0) | Ⅲ(12) | 135 | 0.162(Low) | no | no |
| 166 | obstructive jaundice | 69(5) | Chinese(8) | G2(2) | RA(0) | Elective(0) | High(85) | 16(70) | Ⅲ(12) | 182 | 0.485(High) | yes | no |
| 167 | interstitial tubal pregnancy | 25(10) | Chinese(8) | G1(8) | GA(22) | Emergency(3) | Moderate(52) | 0(0) | Ⅱ(2) | 105 | 0.046(Low) | no | no |
| 168 | cerebral aneurysm | 31(0) | Chinese(8) | G1(8) | GA(22) | Emergency(3) | High(85) | 0(0) | Ⅱ(2) | 128 | 0.092(Low) | no | no |
| 169 | Malignant tumor of skull base | 40(0) | Chinese(8) | G1(8) | GA(22) | Elective(0) | High(85) | 0(0) | Ⅱ(2) | 125 | 0.091(Low) | no | no |
| 170 | gastric cancer | 50(5) | Chinese(8) | G1(8) | GA(22) | Elective(0) | High(85) | 0(0) | Ⅲ(12) | 140 | 0.203(High) | yes | no |
| 171 | malignant tumor | 58(5) | Chinese(8) | G1(8) | GA(22) | Elective(0) | High(85) | 0(0) | Ⅳ(18) | 146 | 0.272(High) | yes | yes |
| 172 | Malignant tumor of skull base | 46(0) | Chinese(8) | G1(8) | GA(22) | Elective(0) | High(85) | 0(0) | Ⅱ(2) | 125 | 0.091(Low) | no | no |
| 173 | adrenal tumor | 27(10) | Chinese(8) | G1(8) | GA(22) | Elective(0) | High(85) | 0(0) | Ⅱ(2) | 135 | 0.123(Low) | no | no |
| 174 | necrotizing pancreatitis | 42(0) | Chinese(8) | G1(8) | GA(22) | Elective(0) | High(85) | 0(0) | Ⅱ(2) | 125 | 0.091(Low) | no | no |
| 175 | Glioma | 42(0) | Chinese(8) | G1(8) | GA(22) | Elective(0) | High(85) | 0(0) | Ⅲ(12) | 135 | 0.162(Low) | no | no |
| 176 | breast cancer | 33(0) | Chinese(8) | G1(8) | RA(0) | Elective(0) | Moderate(52) | 0(0) | Ⅲ(12) | 80 | 0.026(Low) | no | no |
| 177 | trigeminal neuralgia | 52(5) | Chinese(8) | G1(8) | GA(22) | Elective(0) | High(85) | 0(0) | Ⅳ(18) | 146 | 0.272(High) | yes | no |
| 178 | ureteral calculus | 41(0) | Chinese(8) | G2(2) | GA(22) | Elective(0) | High(85) | 0(0) | Ⅱ(2) | 119 | 0.091(Low) | no | no |
| 179 | retroperitoneal mass | 49(0) | Chinese(8) | G1(8) | GA(22) | Elective(0) | High(85) | 0(0) | Ⅲ(12) | 135 | 0.162(Low) | no | no |
| 180 | rectal carcinoma | 63(5) | Chinese(8) | G2(2) | GA(22) | Elective(0) | High(85) | 0(0) | Ⅲ(12) | 134 | 0.203(High) | yes | no |
| 181 | coronary heart disease | 57(5) | Chinese(8) | G1(8) | GA(22) | Elective(0) | High(85) | 0(0) | Ⅳ(18) | 146 | 0.272(High) | yes | no |
| 182 | rectal carcinoma | 59(5) | Chinese(8) | G3(7) | GA(22) | Elective(0) | High(85) | 16(70) | Ⅳ(18) | 215 | 0.776(High) | yes | no |
| 183 | trigeminal neuralgia | 81(12) | Chinese(8) | G3(7) | GA(22) | Elective(0) | High(85) | 17(20) | Ⅲ(12) | 166 | 0.426(High) | yes | no |
| 184 | Space occupying lesion of skull base | 67(5) | Chinese(8) | G1(8) | GA(22) | Elective(0) | High(85) | 0(0) | Ⅱ(2) | 130 | 0.117(Low) | no | no |
| 185 | Intraspinal mass | 43(0) | Chinese(8) | G1(8) | GA(22) | Elective(0) | High(85) | 0(0) | Ⅱ(2) | 125 | 0.091(Low) | no | no |
| 186 | spondylolisthesis | 50(5) | Chinese(8) | G1(8) | GA(22) | Elective(0) | High(85) | 0(0) | Ⅱ(2) | 130 | 0.117(Low) | no | no |
| 187 | duodenal neoplasms | 79(12) | Chinese(8) | G2(2) | GA(22) | Elective(0) | High(85) | 16(70) | Ⅳ(18) | 217 | 0.803(High) | yes | yes |
| 188 | Tongue malignant tumor | 49(0) | Chinese(8) | G1(8) | GA(22) | Elective(0) | High(85) | 0(0) | Ⅲ(12) | 135 | 0.162(Low) | no | no |
| 189 | subdural hematoma | 57(5) | Chinese(8) | G1(8) | RA(0) | Elective(0) | High(85) | 0(0) | Ⅱ(2) | 108 | 0.05(Low) | no | no |
| 190 | Space occupying lesions in saddle region | 67(5) | Chinese(8) | G3(7) | GA(22) | Elective(0) | High(85) | 16(70) | Ⅳ(18) | 215 | 0.776(High) | yes | yes |
| 191 | rectal polyp | 68(5) | Chinese(8) | G2(2) | GA(22) | Elective(0) | Moderate(52) | 0(0) | Ⅱ(2) | 91 | 0.043(Low) | no | no |
| 192 | coronary heart disease | 57(5) | Chinese(8) | G2(2) | GA(22) | Elective(0) | High(85) | 17(20) | Ⅳ(18) | 160 | 0.48(High) | yes | yes |
| 193 | Tongue malignant tumor | 44(0) | Chinese(8) | G1(8) | GA(22) | Elective(0) | High(85) | 0(0) | Ⅲ(12) | 135 | 0.162(Low) | no | no |
| 194 | Space occupying lesion of skull base | 35(0) | Chinese(8) | G2(2) | GA(22) | Elective(0) | High(85) | 0(0) | Ⅲ(12) | 129 | 0.162(Low) | no | no |
| 195 | breast cancer | 75(12) | Chinese(8) | G2(2) | GA(22) | Elective(0) | High(85) | 17(20) | Ⅲ(12) | 161 | 0.426(High) | yes | no |
| 196 | Genital varicose veins | 44(0) | Chinese(8) | G1(8) | RA(0) | Elective(0) | High(85) | 0(0) | Ⅱ(2) | 103 | 0.039(Low) | no | no |
| 197 | mandible tumor | 27(10) | Chinese(8) | G1(8) | GA(22) | Elective(0) | High(85) | 0(0) | Ⅱ(2) | 135 | 0.123(Low) | no | no |
| 198 | rectal carcinoma | 50(5) | Chinese(8) | G1(8) | GA(22) | Elective(0) | High(85) | 11(18) | Ⅲ(12) | 158 | 0.262(High) | yes | no |
| 199 | valvular disease | 40(0) | Chinese(8) | G1(8) | GA(22) | Elective(0) | High(85) | 0(0) | Ⅱ(2) | 125 | 0.091(Low) | no | no |
| 200 | Space occupying lesion of skull base | 53(5) | Chinese(8) | G1(8) | GA(22) | Elective(0) | High(85) | 0(0) | Ⅱ(2) | 130 | 0.117(Low) | no | no |
| 201 | Space occupying lesions in saddle region | 63(5) | Chinese(8) | G1(8) | GA(22) | Elective(0) | High(85) | 0(0) | Ⅲ(12) | 140 | 0.203(High) | yes | no |
| 202 | sacrum malignant tumor | 55(5) | Chinese(8) | G1(8) | GA(22) | Elective(0) | High(85) | 16(70) | Ⅳ(18) | 216 | 0.776(High) | yes | no |
| 203 | hydronephrosis | 67(5) | Chinese(8) | G2(2) | GA(22) | Elective(0) | High(85) | 16(70) | Ⅳ(18) | 210 | 0.776(High) | yes | no |
| 204 | Tongue malignant tumor | 54(5) | Chinese(8) | G1(8) | GA(22) | Elective(0) | High(85) | 0(0) | Ⅲ(12) | 140 | 0.203(High) | yes | no |
| 205 | retroperitoneal tumor | 51(5) | Chinese(8) | G1(8) | GA(22) | Elective(0) | High(85) | 16(70) | Ⅳ(18) | 216 | 0.776(High) | yes | no |
| 206 | facial spasm | 53(5) | Chinese(8) | G1(8) | GA(22) | Elective(0) | High(85) | 0(0) | Ⅲ(12) | 140 | 0.203(High) | yes | no |
| 208 | Space occupying lesion of skull base | 55(5) | Chinese(8) | G2(2) | GA(22) | Elective(0) | High(85) | 0(0) | Ⅲ(12) | 134 | 0.203(High) | yes | no |
| 209 | intracranial space occupying lesion | 28(10) | Chinese(8) | G1(8) | GA(22) | Elective(0) | High(85) | 0(0) | Ⅳ(18) | 151 | 0.282(High) | yes | no |
| 210 | sigmoid colon tumor | 66(5) | Chinese(8) | G1(8) | GA(22) | Elective(0) | High(85) | 0(0) | Ⅲ(12) | 140 | 0.203(High) | yes | no |
| 211 | Femoral deformities | 20(10) | Chinese(8) | G1(8) | GA(22) | Elective(0) | High(85) | 14(78) | Ⅳ(18) | 229 | 0.828(High) | yes | no |
| 212 | valvular disease | 68(5) | Chinese(8) | G4(0) | RA(0) | Elective(0) | High(85) | 0(0) | Ⅲ(12) | 110 | 0.092(Low) | no | no |
| 213 | intracranial space occupying lesion | 35(0) | Chinese(8) | G1(8) | GA(22) | Elective(0) | High(85) | 17(20) | Ⅳ(18) | 161 | 0.411(High) | yes | no |
| 214 | chronic pancreatitis | 41(0) | Chinese(8) | G1(8) | GA(22) | Elective(0) | High(85) | 0(0) | Ⅲ(12) | 135 | 0.162(Low) | no | no |
| 215 | renal calculi | 57(5) | Chinese(8) | G3(7) | GA(22) | Elective(0) | High(85) | 0(0) | Ⅱ(2) | 129 | 0.117(Low) | no | no |
| 216 | Abdominal aortic dissection | 54(5) | Chinese(8) | G1(8) | GA(22) | Elective(0) | High(85) | 0(0) | Ⅲ(12) | 140 | 0.203(High) | yes | no |
| 217 | invasive breast cancer | 54(5) | Chinese(8) | G1(8) | GA(22) | Elective(0) | High(85) | 0(0) | Ⅲ(12) | 140 | 0.203(High) | yes | no |
| 218 | coronary heart disease | 59(5) | Chinese(8) | G1(8) | GA(22) | Elective(0) | High(85) | 0(0) | Ⅲ(12) | 140 | 0.203(High) | yes | no |
| 219 | intracranial space occupying lesion | 29(10) | Chinese(8) | G1(8) | GA(22) | Elective(0) | High(85) | 16(70) | Ⅳ(18) | 221 | 0.784(High) | yes | no |
| 220 | gastric cancer | 67(5) | Chinese(8) | G1(8) | GA(22) | Elective(0) | High(85) | 17(20) | Ⅳ(18) | 166 | 0.48(High) | yes | yes |
| 221 | Glioma | 27(10) | Chinese(8) | G1(8) | GA(22) | Elective(0) | High(85) | 16(70) | Ⅳ(18) | 221 | 0.784(High) | yes | no |
| 222 | transverse colon carcinoma | 72(12) | Chinese(8) | G1(8) | GA(22) | Elective(0) | High(85) | 0(0) | Ⅱ(2) | 137 | 0.136(Low) | no | no |
| 223 | Space occupying lesion of skull base | 44(0) | Chinese(8) | G1(8) | GA(22) | Elective(0) | High(85) | 0(0) | Ⅱ(2) | 125 | 0.091(Low) | no | no |
| 224 | aortic dissection | 59(5) | Chinese(8) | G2(2) | GA(22) | Emergency(3) | High(85) | 0(0) | Ⅱ(2) | 127 | 0.118(Low) | no | no |
| 225 | breast mass | 51(5) | Chinese(8) | G2(2) | GA(22) | Elective(0) | High(85) | 16(70) | Ⅳ(18) | 210 | 0.776(High) | yes | no |
| 226 | Buccal carcinoma | 45(0) | Chinese(8) | G1(8) | GA(22) | Elective(0) | High(85) | 2(76) | Ⅳ(18) | 217 | 0.762(High) | yes | yes |
| 227 | Cervical stenosis | 67(5) | Chinese(8) | G1(8) | GA(22) | Elective(0) | High(85) | 0(0) | Ⅱ(2) | 130 | 0.117(Low) | no | no |
| 228 | Space occupying lesion of skull base | 57(5) | Chinese(8) | G1(8) | GA(22) | Elective(0) | High(85) | 0(0) | Ⅱ(2) | 130 | 0.117(Low) | no | no |
| 229 | sigmoid colon tumor | 52(5) | Chinese(8) | G2(2) | GA(22) | Elective(0) | High(85) | 0(0) | Ⅲ(12) | 134 | 0.203(High) | yes | no |
| 230 | Space occupying lesions in saddle region | 52(5) | Chinese(8) | G1(8) | GA(22) | Elective(0) | High(85) | 17(20) | Ⅳ(18) | 166 | 0.48(High) | yes | no |
| 231 | carcinoma of descending colon | 57(5) | Chinese(8) | G1(8) | GA(22) | Elective(0) | High(85) | 4(22) | Ⅲ(12) | 162 | 0.355(High) | yes | no |
| 232 | Breast nodules | 46(0) | Chinese(8) | G1(8) | GA(22) | Elective(0) | High(85) | 11(16) | Ⅲ(12) | 151 | 0.211(High) | yes | no |
| 233 | facial spasm | 60(5) | Chinese(8) | G2(2) | GA(22) | Elective(0) | High(85) | 17(20) | Ⅲ(12) | 154 | 0.387(High) | yes | no |
| 234 | abdominal tumour | 55(5) | Chinese(8) | G3(7) | GA(22) | Elective(0) | High(85) | 0(0) | Ⅲ(12) | 139 | 0.203(High) | yes | no |
| 235 | Pituitary adenoma | 47(0) | Chinese(8) | G1(8) | GA(22) | Elective(0) | High(85) | 0(0) | Ⅳ(18) | 141 | 0.221(High) | yes | yes |
| 236 | Space occupying lesions in saddle region | 44(0) | Chinese(8) | G1(8) | GA(22) | Elective(0) | High(85) | 0(0) | Ⅳ(18) | 141 | 0.221(High) | yes | yes |
| 237 | Ascending colon tumor | 50(5) | Chinese(8) | G1(8) | GA(22) | Elective(0) | High(85) | 0(0) | Ⅲ(12) | 140 | 0.203(High) | yes | no |
| 238 | Trunk mass | 35(0) | Chinese(8) | G1(8) | GA(22) | Elective(0) | High(85) | 14(78) | Ⅳ(18) | 219 | 0.776(High) | yes | no |
| 239 | acoustic neuroma | 45(0) | Chinese(8) | G1(8) | GA(22) | Elective(0) | High(85) | 0(0) | Ⅱ(2) | 125 | 0.091(Low) | no | no |
| 240 | dentofacial deformity | 21(10) | Chinese(8) | G1(8) | GA(22) | Elective(0) | High(85) | 11(16) | Ⅳ(18) | 167 | 0.353(High) | yes | yes |
| 241 | Buccal carcinoma | 48(0) | Chinese(8) | G2(2) | GA(22) | Elective(0) | High(85) | 0(0) | Ⅱ(2) | 119 | 0.091(Low) | no | no |
| 242 | Oral mass | 51(5) | Chinese(8) | G2(2) | GA(22) | Elective(0) | High(85) | 0(0) | Ⅱ(2) | 124 | 0.117(Low) | no | no |
| 243 | intracranial space occupying lesion | 30(0) | Chinese(8) | G1(8) | GA(22) | Elective(0) | High(85) | 0(0) | Ⅲ(12) | 135 | 0.162(Low) | no | no |
| 244 | intracranial space occupying lesion | 33(0) | Chinese(8) | G2(2) | GA(22) | Elective(0) | High(85) | 4(22) | Ⅳ(18) | 157 | 0.379(High) | yes | no |
| 245 | cervix tumor | 49(0) | Chinese(8) | G1(8) | GA(22) | Elective(0) | High(85) | 0(0) | Ⅲ(12) | 135 | 0.162(Low) | no | no |
| 246 | suppurative otitis media | 50(5) | Chinese(8) | G1(8) | GA(22) | Elective(0) | High(85) | 17(20) | Ⅲ(12) | 160 | 0.387(High) | yes | no |
| 247 | Cholesteatoma of middle ear | 49(0) | Chinese(8) | G1(8) | GA(22) | Elective(0) | High(85) | 0(0) | Ⅲ(12) | 135 | 0.162(Low) | no | no |
| 248 | breast cancer | 58(5) | Chinese(8) | G2(2) | GA(22) | Elective(0) | High(85) | 0(0) | Ⅱ(2) | 124 | 0.117(Low) | no | no |
| 249 | Pituitary adenoma | 59(5) | Chinese(8) | G1(8) | GA(22) | Elective(0) | High(85) | 0(0) | Ⅱ(2) | 130 | 0.117(Low) | no | no |
| 250 | vocal cords hyperplasia | 58(5) | Chinese(8) | G2(2) | GA(22) | Elective(0) | High(85) | 0(0) | Ⅱ(2) | 124 | 0.117(Low) | no | no |
| 251 | colon polyp | 41(0) | Chinese(8) | G1(8) | GA(22) | Elective(0) | Moderate(52) | 17(20) | Ⅳ(18) | 128 | 0.192(High) | yes | no |
| 252 | aortic dissection with IMH | 62(5) | Chinese(8) | G2(2) | GA(22) | Elective(0) | High(85) | 17(20) | Ⅲ(12) | 154 | 0.387(High) | yes | no |
| 253 | Renal dialysis fistula | 33(0) | Chinese(8) | G5(0) | RA(0) | Elective(0) | High(85) | 0(0) | Ⅱ(2) | 95 | 0.039(Low) | no | no |
| 254 | allergic rhinitis | 37(0) | Chinese(8) | G1(8) | GA(22) | Elective(0) | High(85) | 17(20) | Ⅲ(12) | 155 | 0.323(High) | yes | no |
| 255 | meningioma | 48(0) | Chinese(8) | G1(8) | GA(22) | Elective(0) | High(85) | 0(0) | Ⅱ(2) | 125 | 0.091(Low) | no | no |
| 256 | benign parotid tumours | 64(5) | Chinese(8) | G1(8) | GA(22) | Elective(0) | High(85) | 0(0) | Ⅱ(2) | 130 | 0.117(Low) | no | no |
| 257 | pelvic mass | 40(0) | Chinese(8) | G1(8) | GA(22) | Elective(0) | High(85) | 0(0) | Ⅱ(2) | 125 | 0.091(Low) | no | no |
| 258 | cervical intraepithelial neoplasm | 51(5) | Chinese(8) | G1(8) | GA(22) | Elective(0) | Moderate(52) | 0(0) | Ⅱ(2) | 97 | 0.043(Low) | no | no |
| 259 | renal calculi | 47(0) | Chinese(8) | G2(2) | GA(22) | Elective(0) | High(85) | 0(0) | Ⅱ(2) | 119 | 0.091(Low) | no | no |
| 260 | Vertebral artery aneurysms | 53(5) | Chinese(8) | G1(8) | GA(22) | Elective(0) | High(85) | 17(20) | Ⅲ(12) | 160 | 0.387(High) | yes | no |
| 261 | lung squamous carcinoma | 68(5) | Chinese(8) | G2(2) | GA(22) | Elective(0) | High(85) | 0(0) | Ⅱ(2) | 124 | 0.117(Low) | no | no |
| 262 | fetal distress | 37(0) | Chinese(8) | G1(8) | RA(0) | Elective(0) | Moderate(52) | 0(0) | Ⅱ(2) | 70 | 0.014(Low) | no | no |
| 263 | Allergic Rhinitis | 34(0) | Chinese(8) | G1(8) | GA(22) | Elective(0) | High(85) | 17(20) | Ⅲ(12) | 155 | 0.323(High) | yes | no |
| 264 | fetal macrosomia | 26(10) | Chinese(8) | G1(8) | RA(0) | Elective(0) | Moderate(52) | 0(0) | Ⅱ(2) | 80 | 0.019(Low) | no | no |
| 265 | hysteromyoma | 49(0) | Chinese(8) | G1(8) | GA(22) | Elective(0) | High(85) | 0(0) | Ⅱ(2) | 125 | 0.091(Low) | no | no |
| 266 | fetal macrosomia | 31(0) | Chinese(8) | G1(8) | RA(0) | Elective(0) | Moderate(52) | 0(0) | Ⅱ(2) | 70 | 0.014(Low) | no | no |
| 267 | pharyngeala tumor | 39(0) | Chinese(8) | G1(8) | GA(22) | Elective(0) | High(85) | 16(70) | Ⅳ(18) | 211 | 0.724(High) | yes | no |
| 268 | coronary heart disease | 50(5) | Chinese(8) | G1(8) | RA(0) | Elective(0) | High(85) | 0(0) | Ⅲ(12) | 118 | 0.092(Low) | no | no |
| 269 | Sinus mass | 32(0) | Chinese(8) | G1(8) | GA(22) | Elective(0) | High(85) | 0(0) | Ⅲ(12) | 135 | 0.162(Low) | no | no |
| 270 | breast lump | 18(10) | Chinese(8) | G1(8) | GA(22) | Elective(0) | Moderate(52) | 0(0) | Ⅱ(2) | 102 | 0.045(Low) | no | no |
| 271 | Shoulder Injuries and Disorders | 58(5) | Chinese(8) | G1(8) | GA(22) | Elective(0) | High(85) | 16(70) | Ⅳ(18) | 216 | 0.776(High) | yes | no |
| 272 | cesarean delivery with aplastic anemia | 35(0) | Chinese(8) | G1(8) | GA(22) | Elective(0) | Moderate(52) | 6(60) | Ⅳ(18) | 168 | 0.413(High) | yes | yes |
| 273 | hysteromyoma | 48(0) | Chinese(8) | G1(8) | GA(22) | Elective(0) | Moderate(52) | 17(20) | Ⅳ(18) | 128 | 0.192(High) | yes | no |
| 274 | gemellary pregnancy | 32(0) | Chinese(8) | G1(8) | RA(0) | Elective(0) | Moderate(52) | 0(0) | Ⅲ(12) | 80 | 0.026(Low) | no | no |
| 275 | pulmonary bronchogenic cyst | 68(5) | Chinese(8) | G1(8) | GA(22) | Elective(0) | High(85) | 0(0) | Ⅱ(2) | 130 | 0.117(Low) | no | no |
| 276 | Ovarian teratoma | 19(10) | Chinese(8) | G1(8) | GA(22) | Elective(0) | High(85) | 0(0) | Ⅲ(12) | 145 | 0.211(High) | yes | no |
| 277 | fetal distress | 26(10) | Chinese(8) | G2(2) | RA(0) | Elective(0) | High(85) | 0(0) | Ⅱ(2) | 107 | 0.053(Low) | no | no |
| 278 | placenta previa | 28(10) | Chinese(8) | G1(8) | GA(22) | Emergency(3) | High(85) | 17(20) | Ⅲ(12) | 168 | 0.4(High) | yes | no |
| 279 | scar uterus | 38(0) | Chinese(8) | G1(8) | GA(22) | Elective(0) | High(85) | 0(0) | Ⅱ(2) | 125 | 0.091(Low) | no | no |
| 280 | rectal carcinoma | 68(5) | Chinese(8) | G2(2) | GA(22) | Elective(0) | High(85) | 0(0) | Ⅳ(18) | 140 | 0.272(High) | yes | yes |
| 281 | coronary heart disease | 53(5) | Chinese(8) | G2(2) | RA(0) | Elective(0) | High(85) | 2(76) | Ⅲ(12) | 188 | 0.536(High) | yes | no |
| 284 | Ascites | 46(0) | Chinese(8) | G2(2) | GA(22) | Elective(0) | Moderate(52) | 0(0) | Ⅱ(2) | 86 | 0.033(Low) | no | no |
| 285 | parotid gland carcinoma | 68(5) | Chinese(8) | G2(2) | GA(22) | Elective(0) | High(85) | 0(0) | Ⅲ(12) | 134 | 0.203(High) | yes | no |
| 286 | Nasal Polyps | 57(5) | Chinese(8) | G1(8) | GA(22) | Elective(0) | Moderate(52) | 17(20) | Ⅱ(2) | 117 | 0.101(Low) | no | no |
| 287 | rotator cuff injury | 58(5) | Chinese(8) | G1(8) | GA(22) | Elective(0) | High(85) | 17(20) | Ⅳ(18) | 166 | 0.48(High) | yes | no |
| 288 | space-occupying pathologic leision | 48(0) | Chinese(8) | G1(8) | GA(22) | Elective(0) | High(85) | 15(40) | Ⅲ(12) | 175 | 0.371(High) | yes | no |
| 289 | Intestinal anastomotic fistula | 59(5) | Chinese(8) | G2(2) | GA(22) | Elective(0) | High(85) | 0(0) | Ⅱ(2) | 124 | 0.117(Low) | no | no |
| 290 | intracranial space occupying lesion | 59(5) | Chinese(8) | G1(8) | GA(22) | Elective(0) | High(85) | 0(0) | Ⅱ(2) | 130 | 0.117(Low) | no | no |
| 291 | Tonsil cancer | 53(5) | Chinese(8) | G2(2) | GA(22) | Elective(0) | High(85) | 0(0) | Ⅱ(2) | 124 | 0.117(Low) | no | no |
| 292 | inguinal hernia | 77(12) | Chinese(8) | G1(8) | GA(22) | Elective(0) | High(85) | 0(0) | Ⅲ(12) | 147 | 0.231(High) | yes | no |
| 293 | cerebral aneurysm | 52(5) | Chinese(8) | G2(2) | GA(22) | Elective(0) | High(85) | 0(0) | Ⅲ(12) | 134 | 0.203(High) | yes | no |
| 294 | lung nodules | 56(5) | Chinese(8) | G2(2) | GA(22) | Elective(0) | High(85) | 0(0) | Ⅲ(12) | 134 | 0.203(High) | yes | no |
| 295 | paroxysmal atrial fibrillation | 53(5) | Chinese(8) | G2(2) | GA(22) | Elective(0) | High(85) | 17(20) | Ⅲ(12) | 154 | 0.387(High) | yes | no |
| 296 | rectal carcinoma | 67(5) | Chinese(8) | G1(8) | GA(22) | Elective(0) | High(85) | 17(20) | Ⅳ(18) | 166 | 0.48(High) | yes | no |
| 297 | calculus of bile duct | 54(5) | Chinese(8) | G1(8) | GA(22) | Elective(0) | High(85) | 17(20) | Ⅲ(12) | 160 | 0.387(High) | yes | no |
| 298 | acoustic neuroma | 66(5) | Chinese(8) | G1(8) | GA(22) | Elective(0) | High(85) | 0(0) | Ⅱ(2) | 130 | 0.117(Low) | no | no |
| 299 | Tongue malignant tumor | 57(5) | Chinese(8) | G3(7) | GA(22) | Elective(0) | High(85) | 13(18) | Ⅳ(18) | 163 | 0.403(High) | yes | yes |
| 300 | rectal carcinoma | 50(5) | Chinese(8) | G2(2) | GA(22) | Elective(0) | Moderate(52) | 0(0) | Ⅱ(2) | 91 | 0.043(Low) | no | no |
| 301 | pituitary tumor | 26(10) | Chinese(8) | G1(8) | GA(22) | Elective(0) | High(85) | 2(76) | Ⅲ(12) | 221 | 0.752(High) | yes | no |
| 302 | Renal space occupying lesion | 74(12) | Chinese(8) | G3(7) | GA(22) | Elective(0) | High(85) | 13(18) | Ⅳ(18) | 170 | 0.443(High) | yes | yes |
| 303 | sigmoid colon tumor | 32(0) | Chinese(8) | G1(8) | GA(22) | Elective(0) | High(85) | 16(70) | Ⅲ(12) | 205 | 0.641(High) | yes | no |
| 304 | space-occupying pathologic leision | 75(12) | Chinese(8) | G3(7) | GA(22) | Elective(0) | High(85) | 0(0) | Ⅲ(12) | 146 | 0.231(High) | yes | no |
| 305 | intracranial space occupying lesion | 33(0) | Chinese(8) | G1(8) | GA(22) | Elective(0) | High(85) | 0(0) | Ⅱ(2) | 125 | 0.091(Low) | no | no |
| 306 | wound separation | 55(5) | Chinese(8) | G3(7) | GA(22) | Elective(0) | Moderate(52) | 0(0) | Ⅱ(2) | 96 | 0.043(Low) | no | no |
| 307 | anterior cruciate ligament injury | 28(10) | Chinese(8) | G1(8) | RA(0) | Elective(0) | High(85) | 16(70) | Ⅲ(12) | 193 | 0.497(High) | yes | no |
| 308 | Space occupying lesions in bladder | 67(5) | Chinese(8) | G4(0) | GA(22) | Elective(0) | High(85) | 16(70) | Ⅲ(12) | 202 | 0.702(High) | yes | no |
| 309 | hamartoma of kidney | 37(0) | Chinese(8) | G1(8) | GA(22) | Elective(0) | High(85) | 0(0) | Ⅱ(2) | 125 | 0.091(Low) | no | no |
| 310 | palpitation. | 57(5) | Chinese(8) | G1(8) | GA(22) | Elective(0) | High(85) | 0(0) | Ⅱ(2) | 130 | 0.117(Low) | no | no |
| 311 | lumbar spinal stenosis | 51(5) | Chinese(8) | G1(8) | GA(22) | Elective(0) | High(85) | 0(0) | Ⅱ(2) | 130 | 0.117(Low) | no | no |
| 312 | acute pancreatitis | 50(5) | Chinese(8) | G1(8) | GA(22) | Elective(0) | High(85) | 0(0) | Ⅲ(12) | 140 | 0.203(High) | yes | no |
| 313 | hepatolithiasis | 69(5) | Chinese(8) | G2(2) | GA(22) | Elective(0) | High(85) | 17(20) | Ⅲ(12) | 154 | 0.387(High) | yes | no |
| 314 | sprained ankle | 19(10) | Chinese(8) | G1(8) | GA(22) | Elective(0) | High(85) | 0(0) | Ⅲ(12) | 145 | 0.211(High) | yes | no |
| 315 | mass of skull base | 45(0) | Chinese(8) | G1(8) | GA(22) | Elective(0) | High(85) | 16(70) | Ⅳ(18) | 211 | 0.724(High) | yes | no |
| 316 | cervical spondylosis | 48(0) | Chinese(8) | G1(8) | GA(22) | Elective(0) | High(85) | 0(0) | Ⅲ(12) | 135 | 0.162(Low) | no | no |
| 317 | lumbar spinal stenosis | 59(5) | Chinese(8) | G1(8) | GA(22) | Elective(0) | High(85) | 2(76) | Ⅳ(18) | 222 | 0.809(High) | yes | no |
| 318 | acute cholangitis | 70(12) | Chinese(8) | G1(8) | GA(22) | Elective(0) | High(85) | 0(0) | Ⅱ(2) | 137 | 0.136(Low) | no | no |
| 319 | Oral Submucous Fibrosis | 47(0) | Chinese(8) | G2(2) | GA(22) | Elective(0) | High(85) | 17(20) | Ⅲ(12) | 149 | 0.323(High) | yes | no |
| 320 | mandible tumor | 36(0) | Chinese(8) | G1(8) | GA(22) | Elective(0) | Moderate(52) | 0(0) | Ⅱ(2) | 92 | 0.033(Low) | no | no |
| 321 | femoral neck fractures | 57(5) | Chinese(8) | G1(8) | GA(22) | Elective(0) | High(85) | 0(0) | Ⅱ(2) | 130 | 0.117(Low) | no | no |
| 322 | laryngeal pathological change | 64(5) | Chinese(8) | G1(8) | GA(22) | Elective(0) | High(85) | 0(0) | Ⅲ(12) | 140 | 0.203(High) | yes | no |
| 323 | lung cancer | 40(0) | Chinese(8) | G2(2) | GA(22) | Elective(0) | High(85) | 0(0) | Ⅲ(12) | 129 | 0.162(Low) | no | no |
| 324 | Ruptured extensor finger | 52(5) | Chinese(8) | G3(7) | GA(22) | Elective(0) | High(85) | 16(70) | Ⅳ(18) | 215 | 0.776(High) | yes | no |
| 325 | aortic valve stenosis | 72(12) | Chinese(8) | G1(8) | GA(22) | Elective(0) | High(85) | 0(0) | Ⅱ(2) | 137 | 0.136(Low) | no | no |
| 326 | intracranial space occupying lesion | 21(10) | Chinese(8) | G1(8) | GA(22) | Elective(0) | High(85) | 15(40) | Ⅲ(12) | 185 | 0.45(High) | yes | no |
| 327 | femoral neck fractures | 46(0) | Chinese(8) | G1(8) | GA(22) | Elective(0) | High(85) | 0(0) | Ⅱ(2) | 125 | 0.091(Low) | no | no |
| 328 | meniscus injury | 53(5) | Chinese(8) | G2(2) | GA(22) | Elective(0) | High(85) | 16(70) | Ⅳ(18) | 210 | 0.776(High) | yes | no |
| 329 | Parotid gland mass | 33(0) | Chinese(8) | G1(8) | GA(22) | Elective(0) | High(85) | 0(0) | Ⅱ(2) | 125 | 0.091(Low) | no | no |
| 330 | valvular disease | 44(0) | Chinese(8) | G1(8) | GA(22) | Elective(0) | High(85) | 4(22 ) | Ⅲ(12) | 157 | 0.294(High) | yes | no |
| 331 | Crohn's disease | 20(10) | Chinese(8) | G1(8) | GA(22) | Elective(0) | Moderate(52) | 0(0) | Ⅱ(2) | 102 | 0.045(Low) | no | no |
| 332 | lumbar spinal stenosis | 48(0) | Chinese(8) | G1(8) | GA(22) | Elective(0) | High(85) | 0(0) | Ⅱ(2) | 125 | 0.091(Low) | no | no |
| 333 | maxilla mass | 67(5) | Chinese(8) | G1(8) | GA(22) | Elective(0) | Moderate(52) | 0(0) | Ⅱ(2) | 97 | 0.043(Low) | no | no |
| 334 | cholelithiasis | 34(0) | Chinese(8) | G1(8) | GA(22) | Elective(0) | High(85) | 2(76) | Ⅲ(12) | 211 | 0.686(High) | yes | no |
| 335 | thyroid nodule | 30(0) | Chinese(8) | G1(8) | GA(22) | Elective(0) | High(85) | 0(0) | Ⅱ(2) | 125 | 0.091(Low) | no | no |
| 336 | meniscus injury | 79(12) | Chinese(8) | G2(2) | GA(22) | Elective(0) | High(85) | 0(0) | Ⅱ(2) | 131 | 0.136(Low) | no | no |
| 337 | thyroid nodule | 49(0) | Chinese(8) | G1(8) | GA(22) | Elective(0) | High(85) | 13(18) | Ⅲ(12) | 153 | 0.258(High) | yes | no |
| 338 | chronic suppurative otitis media | 49(0) | Chinese(8) | G2(2) | GA(22) | Elective(0) | High(85) | 0(0) | Ⅲ(12) | 129 | 0.162(Low) | no | no |
| 339 | ureterostenosis | 50(5) | Chinese(8) | G2(2) | GA(22) | Elective(0) | High(85) | 13(18) | Ⅲ(12) | 152 | 0.315(High) | yes | no |
| 340 | valvular disease | 72(12) | Chinese(8) | G2(2) | GA(22) | Elective(0) | High(85) | 17(20) | Ⅳ(18) | 167 | 0.521(High) | yes | yes |
| 341 | lumbar spinal stenosis | 62(5) | Chinese(8) | G1(8) | GA(22) | Elective(0) | High(85) | 0(0) | Ⅱ(2) | 206 | 0.117(Low) | no | no |
| 342 | intracranial space occupying lesion | 43(0) | Chinese(8) | G1(8) | GA(22) | Elective(0) | High(85) | 2(76) | Ⅳ(18) | 141 | 0.762(High) | yes | no |
| 343 | intracranial space occupying lesion | 60(5) | Chinese(8) | G1(8) | GA(22) | Elective(0) | High(85) | 17(20) | Ⅳ(18) | 166 | 0.48(High) | yes | yes |
| 344 | malocclusion | 20(10) | Chinese(8) | G1(8) | GA(22) | Elective(0) | High(85) | 0(0) | Ⅳ(18) | 151 | 0.282(High) | yes | yes |
| 345 | Skin mass | 34(0) | Chinese(8) | G2(2) | GA(22) | Elective(0) | High(85) | 0(0) | Ⅱ(2) | 119 | 0.091(Low) | no | no |
| 346 | breast cancer | 59(5) | Chinese(8) | G2(2) | GA(22) | Elective(0) | High(85) | 0(0) | Ⅲ(12) | 134 | 0.203(High) | yes | no |
| 347 | paroxysmal supraventricular tachycardia | 52(5) | Chinese(8) | G1(8) | RA(0) | Elective(0) | High(85) | 15(40) | Ⅲ(12) | 158 | 0.237(High) | yes | no |
| 348 | lower extremity atherosclerotic occlusive disease | 57(5) | Chinese(8) | G5(0) | GA(22) | Elective(0) | High(85) | 0(0) | Ⅱ(2) | 122 | 0.117(Low) | no | no |
| 349 | colon carcinoma | 34(0) | Chinese(8) | G1(8) | GA(22) | Elective(0) | High(85) | 0(0) | Ⅱ(2) | 125 | 0.091(Low) | no | no |
| 350 | oropharynx malignant tumor | 49(0) | Chinese(8) | G2(2) | GA(22) | Elective(0) | High(85) | 14(78) | Ⅳ(18) | 213 | 0.776(High) | yes | no |
| 351 | gastric cancer | 37(0) | Chinese(8) | G1(8) | GA(22) | Elective(0) | High(85) | 17(20) | Ⅲ(12) | 155 | 0.323(High) | yes | no |
| 352 | pulmonary nodules | 62(5) | Chinese(8) | G1(8) | GA(22) | Elective(0) | High(85) | 0(0) | Ⅱ(2) | 130 | 0.117(Low) | no | no |
| 353 | breast cancer | 33(0) | Chinese(8) | G1(8) | GA(22) | Elective(0) | High(85) | 17(20) | Ⅲ(12) | 155 | 0.323(High) | yes | no |
| 354 | Space occupying lesions in bladder | 59(5) | Chinese(8) | G2(2) | GA(22) | Elective(0) | High(85) | 0(0) | Ⅱ(2) | 124 | 0.117(Low) | no | no |
| 355 | lumbar intervertebral disc hernia | 21(10) | Chinese(8) | G1(8) | GA(22) | Elective(0) | High(85) | 17(20) | Ⅲ(12) | 165 | 0.398(High) | yes | no |
| 356 | impacted tooth | 23(10) | Chinese(8) | G1(8) | GA(22) | Elective(0) | Moderate(52) | 0(0) | Ⅱ(2) | 102 | 0.045(Low) | no | no |
| 357 | internal carotid artery aneurysm | 60(5) | Chinese(8) | G2(2) | GA(22) | Elective(0) | High(85) | 0(0) | Ⅱ(2) | 124 | 0.117(Low) | no | no |
| 358 | Ventricular chondrosarcoma | 53(5) | Chinese(8) | G1(8) | GA(22) | Elective(0) | High(85) | 0(0) | Ⅱ(2) | 130 | 0.117(Low) | no | no |
| 359 | lower limb tumor | 47(0) | Chinese(8) | G1(8) | GA(22) | Elective(0) | Moderate(52) | 17(20) | Ⅲ(12) | 122 | 0.14(Low) | no | no |
| 360 | Malignant tumor of prostate | 64(5) | Chinese(8) | G1(8) | GA(22) | Elective(0) | High(85) | 17(20) | Ⅲ(12) | 160 | 0.387(High) | yes | no |
| 361 | scar ulcer | 76(12) | Chinese(8) | G2(2) | GA(22) | Elective(0) | High(85) | 0(0) | Ⅱ(2) | 131 | 0.136(Low) | no | no |
| 362 | Foreign bodies remain | 51(5) | Chinese(8) | G2(2) | GA(22) | Elective(0) | High(85) | 17(20) | Ⅲ(12) | 154 | 0.387(High) | yes | no |
| 363 | varicose veins of lower limb | 53(5) | Chinese(8) | G1(8) | GA(22) | Elective(0) | High(85) | 17(20) | Ⅲ(12) | 160 | 0.387(High) | yes | no |
| 364 | meningioma | 44(0) | Chinese(8) | G1(8) | GA(22) | Elective(0) | High(85) | 0(0) | Ⅲ(12) | 135 | 0.162(Low) | no | no |
| 365 | fracture of fibula | 48(0) | Chinese(8) | G1(8) | GA(22) | Elective(0) | Low(0) | 0(0) | Ⅳ(18) | 56 | 0.024(Low) | no | no |
| 366 | lymphedema | 55(5) | Chinese(8) | G1(8) | GA(22) | Elective(0) | High(85) | 0(0) | Ⅲ(12) | 140 | 0.203(High) | yes | no |
| 367 | spinal arteriovenous malformations | 61(5) | Chinese(8) | G2(2) | RA(0) | Elective(0) | Low(0) | 17(20) | Ⅲ(12) | 47 | 0.021(Low) | no | no |
| 368 | Flap bloated | 53(5) | Chinese(8) | G1(8) | GA(22) | Elective(0) | High(85) | 17(20) | Ⅲ(12) | 160 | 0.387(High) | yes | no |
| 369 | hypermastia | 22(10) | Chinese(8) | G1(8) | GA(22) | Elective(0) | High(85) | 0(0) | Ⅱ(2) | 135 | 0.123(Low) | no | no |
| 370 | urethrostenosis | 56(5) | Chinese(8) | G1(8) | GA(22) | Elective(0) | High(85) | 16(70) | Ⅲ(12) | 210 | 0.702(High) | yes | no |
| 371 | Space occupying lesions in bladder | 63(5) | Chinese(8) | G1(8) | GA(22) | Elective(0) | High(85) | 0(0) | Ⅱ(2) | 130 | 0.117(Low) | no | no |
| 372 | Space occupying lesions in bladder | 43(0) | Chinese(8) | G1(8) | GA(22) | Elective(0) | High(85) | 0(0) | Ⅱ(2) | 125 | 0.091(Low) | no | no |
| 373 | papillary thyroid carcinoma | 42(0) | Chinese(8) | G1(8) | GA(22) | Elective(0) | High(85) | 14(78) | Ⅲ(12) | 213 | 0.703(High) | yes | no |
| 374 | Intraspinal mass | 34(0) | Chinese(8) | G1(8) | GA(22) | Elective(0) | High(85) | 0(0) | Ⅱ(2) | 125 | 0.091(Low) | no | no |
| 375 | lung cancer | 67(5) | Chinese(8) | G1(8) | GA(22) | Elective(0) | High(85) | 0(0) | Ⅱ(2) | 130 | 0.117(Low) | no | no |
| 376 | rectal prolapse | 53(5) | Chinese(8) | G1(8) | GA(22) | Elective(0) | Moderate(52) | 0(0) | Ⅲ(12) | 107 | 0.08(Low) | no | no |
| 377 | hemangioma | 65(5) | Chinese(8) | G1(8) | GA(22) | Elective(0) | High(85) | 14(78) | Ⅲ(12) | 218 | 0.757(High) | yes | no |
| 378 | Budd-Chiari's syndrome | 68(5) | Chinese(8) | G2(2) | RA(0) | Elective(0) | High(85) | 0(0) | Ⅱ(2) | 102 | 0.05(Low) | no | no |
| 379 | papillary thyroid carcinoma | 37(0) | Chinese(8) | G1(8) | GA(22) | Elective(0) | High(85) | 14(78) | Ⅲ(12) | 213 | 0.703(High) | yes | no |
| 380 | gastric cancer | 62(5) | Chinese(8) | G1(8) | GA(22) | Elective(0) | High(85) | 17(20) | Ⅳ(18) | 166 | 0.48(High) | yes | yes |
| 381 | intracranial space occupying lesion | 54(5) | Chinese(8) | G1(8) | GA(22) | Elective(0) | High(85) | 0(0) | Ⅳ(18) | 146 | 0.272(High) | yes | yes |
| 382 | meningioma | 57(5) | Chinese(8) | G1(8) | GA(22) | Elective(0) | High(85) | 0(0) | Ⅲ(12) | 140 | 0.203(High) | yes | no |
| 383 | thyroid papillary carcinoma | 41(0) | Chinese(8) | G1(8) | GA(22) | Elective(0) | High(85) | 16(70) | Ⅲ(12) | 205 | 0.641(High) | yes | no |
| 384 | bladder cancer | 57(5) | Chinese(8) | G1(8) | GA(22) | Elective(0) | High(85) | 0(0) | Ⅲ(12) | 140 | 0.203(High) | yes | no |
| 385 | intracranial space occupying lesion | 25(10) | Chinese(8) | G1(8) | GA(22) | Elective(0) | High(85) | 17(20) | Ⅲ(12) | 165 | 0.398(High) | yes | no |
| 386 | Renal space occupying lesion | 58(5) | Chinese(8) | G2(2) | GA(22) | Elective(0) | High(85) | 16(70) | Ⅱ(2) | 194 | 0.552(High) | yes | no |
| 387 | facial spasm | 48(0) | Chinese(8) | G1(8) | GA(22) | Elective(0) | High(85) | 0(0) | Ⅱ(2) | 125 | 0.091(Low) | no | no |
| 388 | metastaic carcinoma of lymph node | 32(0) | Chinese(8) | G1(8) | GA(22) | Elective(0) | High(85) | 0(0) | Ⅲ(12) | 135 | 0.162(Low) | no | no |
| 389 | thyroid caricinoma | 64(5) | Chinese(8) | G1(8) | GA(22) | Elective(0) | High(85) | 17(20) | Ⅲ(12) | 160 | 0.387(High) | yes | no |
| 390 | Space occupying lesions in saddle region | 32(0) | Chinese(8) | G1(8) | GA(22) | Elective(0) | High(85) | 7(38) | Ⅲ(12) | 173 | 0.366(High) | yes | no |
| 391 | perimary hepatic carcinoma | 64(5) | Chinese(8) | G1(8) | RA(0) | Elective(0) | Moderate(52) | 17(20) | Ⅲ(12) | 105 | 0.079(Low) | no | no |
| 392 | Renal space occupying lesion | 59(5) | Chinese(8) | G2(2) | GA(22) | Elective(0) | High(85) | 0(0) | Ⅱ(2) | 124 | 0.117(Low) | no | no |
| 393 | tongue mass | 50(5) | Chinese(8) | G2(2) | GA(22) | Elective(0) | Low(0) | 0(0) | Ⅲ(12) | 49 | 0.021(Low) | no | no |
| 394 | varicose veins of lower limb | 54(5) | Chinese(8) | G1(8) | RA(0) | Elective(0) | High(85) | 14(78) | Ⅳ(18) | 202 | 0.646(High) | yes | no |
| 395 | Liver space-occupying lesion | 47(0) | Chinese(8) | G1(8) | GA(22) | Elective(0) | High(85) | 0(0) | Ⅲ(12) | 135 | 0.162(Low) | no | no |
| 396 | Liver space-occupying lesion | 32(0) | Chinese(8) | G1(8) | GA(22) | Elective(0) | High(85) | 0(0) | Ⅲ(12) | 135 | 0.162(Low) | no | no |
| 397 | thyroid caricinoma | 44(0) | Chinese(8) | G2(2) | GA(22) | Elective(0) | High(85) | 0(0) | Ⅱ(2) | 119 | 0.091(Low) | no | no |
| 398 | Liver space-occupying lesion | 28(10) | Chinese(8) | G1(8) | GA(22) | Elective(0) | High(85) | 2(76) | Ⅳ(18) | 227 | 0.817(High) | yes | no |
| 399 | hemangioma | 39(0) | Chinese(8) | G1(8) | GA(22) | Elective(0) | High(85) | 0(0) | Ⅲ(12) | 135 | 0.162(Low) | no | no |
| 400 | thyroid caricinoma | 37(0) | Chinese(8) | G1(8) | GA(22) | Elective(0) | High(85) | 0(0) | Ⅲ(12) | 135 | 0.162(Low) | no | no |
| 401 | lung adenocarcinoma | 53(5) | Chinese(8) | G1(8) | GA(22) | Elective(0) | High(85) | 0(0) | Ⅲ(12) | 140 | 0.203(High) | yes | no |
| 402 | rectal carcinoma | 60(5) | Chinese(8) | G1(8) | GA(22) | Elective(0) | High(85) | 0(0) | Ⅱ(2) | 130 | 0.117(Low) | no | no |
| 403 | gastric cancer | 69(5) | Chinese(8) | G2(2) | GA(22) | Elective(0) | Moderate(52) | 0(0) | Ⅲ(12) | 101 | 0.08(Low) | no | no |
| 404 | lung adenocarcinoma | 68(5) | Chinese(8) | G2(2) | GA(22) | Elective(0) | High(85) | 0(0) | Ⅱ(2) | 124 | 0.117(Low) | no | no |
| 405 | brainstem cavernous malformation | 42(0) | Chinese(8) | G1(8) | GA(22) | Elective(0) | High(85) | 17(20) | Ⅲ(12) | 155 | 0.323(High) | yes | no |
| 406 | renal tuberculosis | 57(5) | Chinese(8) | G3(7) | GA(22) | Elective(0) | Moderate(52) | 0(0) | Ⅱ(2) | 96 | 0.043(Low) | no | no |
| 407 | adrenocortical nodule | 38(0) | Chinese(8) | G1(8) | GA(22) | Elective(0) | High(85) | 0(0) | Ⅱ(2) | 125 | 0.091(Low) | no | no |
| 408 | renal calculi | 53(5) | Chinese(8) | G2(2) | GA(22) | Elective(0) | High(85) | 0(0) | Ⅱ(2) | 124 | 0.117(Low) | no | no |
| 409 | hippocampal sclerosis | 32(0) | Chinese(8) | G1(8) | GA(22) | Elective(0) | High(85) | 0(0) | Ⅱ(2) | 125 | 0.091(Low) | no | no |
| 64 | Leukoplakia | 51(5) | Chinese(8) | G2(2) | GA(22) | Elective(0) | High(85) | 10(3) | Ⅱ(2) | 127 | 0.139(Low) | no | no |
| 403 | gastric cancer | 48(0) | Chinese(8) | G2(2) | GA(22) | Elective(0) | Moderate(52) | 0(0) | Ⅲ(12) | 96 | 0.062(Low) | no | no |
| 335 | thyroid nodule | 53(5) | Chinese(8) | G1(8) | GA(22) | Elective(0) | High(85) | 0(0) | Ⅱ(2) | 130 | 0.117(Low) | no | no |
| 116 | ovarian cancer | 51(5) | Chinese(8) | G1(8) | GA(22) | Elective(0) | High(85) | 0(0) | Ⅲ(12) | 140 | 0.203(High) | yes | no |
| 375 | lung cancer | 62(5) | Chinese(8) | G1(8) | GA(22) | Elective(0) | High(85) | 0(0) | Ⅱ(2) | 130 | 0.117(Low) | no | no |
| 332 | lumbar spinal stenosis | 58(5) | Chinese(8) | G1(8) | GA(22) | Elective(0) | High(85) | 0(0) | Ⅱ(2) | 130 | 0.117(Low) | no | no |
| 352 | pulmonary nodules | 52(5) | Chinese(8) | G1(8) | GA(22) | Elective(0) | High(85) | 0(0) | Ⅱ(2) | 130 | 0.117(Low) | no | no |
