# Supplementary material for: A novel model forecasting perioperative red blood cell transfusion
Source: Sci Rep. 2022 Sep 27;12:16127. doi: 10.1038/s41598-022-20543-7 (PMC9514715; doi:10.1038/s41598-022-20543-7)
Supplement: Supplementary file 5 — Supplementary Information 4. [file 41598_2022_20543_MOESM5_ESM.docx]

**Table 5S ROC analysis for transfusion risk.**

| **Test** | **n/N** | **ROC area**  **(AUC)** | **95%CI low** | **95%CI upp** | **Cut-off**  **value** | **Specificity** | **Sensitivity** | **Accuracy** |
| --- | --- | --- | --- | --- | --- | --- | --- | --- |
| Total points | 59/400 | 0.857 | 0.817 | 0.897 | 140.5 | 0.683 | 0.932 | 0.720 |

n, numbers of transfusion cases; N, sample size.

**Table 6S ROC analysis of each variable for transfusion risk.**

|  | **AUC** | **Sensitivity** | **Specificity** | **Positive pv** | **Negative pv** | **Accuracy** | **Positive-LR** | **Negative-LR** |
| --- | --- | --- | --- | --- | --- | --- | --- | --- |
| Surgery Risk | 0.741 | 0.8906 | 0.517 | 0.106 | 0.9866 | 0.5396 | 1.8441 | 0.2115 |
| Race | 0.517 | 0.839 | 0.1913 | 0.0625 | 0.9486 | 0.2304 | 1.0374 | 0.8418 |
| age | 0.603 | 0.7339 | 0.4129 | 0.0744 | 0.9602 | 0.4323 | 1.2502 | 0.6443 |
| Type of anesthesia | / | 0.0958 | 0.8488 | 0.0391 | 0.9359 | 0.8033 | 0.6335 | 1.0653 |
| Grade of Kidney disease | 0.571 | 0.207 | 0.9107 | 0.1297 | 0.947 | 0.8681 | 2.3169 | 0.8708 |
| Priority | / | 0.2568 | 0.8047 | 0.078 | 0.9439 | 0.7716 | 1.3149 | 0.9236 |
| ASA | 0.662 | 0.4534 | 0.8113 | 0.1339 | 0.9585 | 0.7897 | 2.4029 | 0.6738 |
| 18 level variable | 0.762 | 0.6399 | 0.7949 | 0.1671 | 0.9717 | 0.7856 | 3.1205 | 0.4529 |
| Anaemia | 0.748 | 0.6773 | 0.7616 | 0.1545 | 0.9735 | 0.7565 | 2.8409 | 0.4237 |
| MCV | 0.56 | 0.2283 | 0.8923 | 0.12 | 0.9473 | 0.8522 | 2.1201 | 0.8649 |
| RDW | / | 0.2862 | 0.9197 | 0.1865 | 0.9525 | 0.8814 | 3.5657 | 0.7761 |
